# Molecular mechanism of the allosteric regulation of the αγ heterodimer of human NAD-dependent isocitrate dehydrogenase

**DOI:** 10.1038/srep40921

**Published:** 2017-01-18

**Authors:** Tengfei Ma, Yingjie Peng, Wei Huang, Jianping Ding

**Affiliations:** 1 National Center for Protein Science Shanghai, State Key Laboratory of Molecular Biology, Center for Excellence in Molecular Cell Science, Institute of Biochemistry and Cell Biology, Shanghai Institutes for Biological Sciences, Chinese Academy of Sciences, 320 Yueyang Road, Shanghai 200031, China; 2 School of Life Science and Technology, ShanghaiTech University, 100 Haike Road, Shanghai 201210, China; 3 Shanghai Science Research Center, Chinese Academy of Sciences, 333 Haike Road, Shanghai 201210, China; 4 Collaborative Innovation Center for Genetics and Development, Fudan University, 2005 Songhu Road, Shanghai 200438, China

## Abstract

Human NAD-dependent isocitrate dehydrogenase catalyzes the decarboxylation of isocitrate (ICT) into α-ketoglutarate in the Krebs cycle. It exists as the α_2_βγ heterotetramer composed of the αβ and αγ heterodimers. Previously, we have demonstrated biochemically that the α_2_βγ heterotetramer and αγ heterodimer can be allosterically activated by citrate (CIT) and ADP. In this work, we report the crystal structures of the αγ heterodimer with the γ subunit bound without or with different activators. Structural analyses show that CIT, ADP and Mg^2+^ bind adjacent to each other at the allosteric site. The CIT binding induces conformational changes at the allosteric site, which are transmitted to the active site through the heterodimer interface, leading to stabilization of the ICT binding at the active site and thus activation of the enzyme. The ADP binding induces no further conformational changes but enhances the CIT binding through Mg^2+^-mediated interactions, yielding a synergistic activation effect. ICT can also bind to the CIT-binding subsite, which induces similar conformational changes but exhibits a weaker activation effect. The functional roles of the key residues are verified by mutagenesis, kinetic and structural studies. Our structural and functional data together reveal the molecular mechanism of the allosteric regulation of the αγ heterodimer.

Isocitrate dehydrogenases (IDHs) are a family of enzymes that catalyze the oxidative decarboxylation of isocitrate (ICT) into α-ketoglutarate (α-KG) using NADP or NAD as coenzyme. Most bacteria and archaea contain only NADP-dependent IDHs (NADP-IDHs, EC 1.1.1.42) in the cytosol, which carry out the catalytic reaction in the Krebs or tricarboxylic acid (TCA) cycle. Eukaryotes contain both NADP-IDHs and NAD-dependent IDHs (NAD-IDHs, EC 1.1.1.41). The eukaryotic NAD-IDHs, located in the mitochondria, exert the catalytic activity in the Krebs cycle. The eukaryotic NADP-IDHs, located in the cytosol, mitochondria and peroxisomes, are demonstrated to play important roles in cellular defense against oxidative damage, detoxification of reactive oxygen species, and synthesis of fat and cholesterol^1^,^2^,^3^,^4^,^5^,^6^,^7^. In particular, human cytosolic and mitochondrial NADP-IDHs (also called IDH1 and IDH2) have been implicated in oncogenesis of tumors. Mutations of human IDH1 and IDH2 have been identified in multiple types of tumors and the mutant proteins confer new function to convert α-KG into 2-hydroxyglutarate (2-HG) whose accumulation can result in altered metabolism and epigenetic dysregulation, leading to genesis and development of cancers^7^,^8^,^9^,^10^,^11^,^12^.

The molecular mechanisms of the function and catalytic reaction of NADP-IDHs have been extensively studied at the biochemical and structural levels. The crystal structures of NADP-IDHs from various species, including *E. coli* NADP-IDH (EcIDH), porcine mitochondrial NADP-IDH (PmIDH) and human cytosolic NADP-IDH (HcIDH) have been determined^13^,^14^,^15^,^16^. All of these enzymes exist and function as homodimers and share a conserved catalytic mechanism, but appear to have different regulatory mechanisms. The activity of EcIDH is regulated by a bi-functional kinase/phosphatase, namely AceK, through reversible phosphorylation of Ser113 at the active site^17^,^18^. The activity of HcIDH seems to be regulated through conformational changes of the active site upon the substrate binding^9^,^14^.

NAD-IDHs are more complex than NADP-IDHs in both composition and regulation. Different from NADP-IDHs, yeast NAD-IDH is composed of a regulatory subunit IDH1 and a catalytic subunit IDH2, which form a heterodimer that is assembled into a heterotetramer and further into a heterooctamer^19^,^20^,^21^,^22^,^23^,^24^,^25^,^26^. IDH1 contains the binding sites for the regulators citrate (CIT) and AMP, and IDH2 contains the binding sites for the substrate ICT and coenzyme NAD. The crystal structure of *Saccharomyces cerevisiae* NAD-IDH has been reported, which shows that the binding of CIT and AMP at the allosteric site could induce conformational changes of the active site and thus enhances the binding affinity for ICT^21^.

Mammalian NAD-IDHs are even more complex than yeast NAD-IDH. These enzymes are composed of three types of subunits in the ratio of 2α:1β:1γ^27^, which share about 40–52% sequence identity^28^,^29^. The α and β subunits form one heterodimer (αβ) and the α and γ subunits form another heterodimer (αγ), which are assembled into a heterotetramer (α_2_βγ) and further into a heterooctamer (the heterotetramer and heterooctamer are sometimes called holoenzyme)^30^,^31^. The previous biochemical data showed that the α subunit is essential for the catalytic activity whereas the β and γ subunits play regulatory roles in the α_2_βγ heterotetramer, and the activity of the α_2_βγ heterotetramer is positively regulated by CIT and ADP but inhibited by ATP and NADH^32^,^33^,^34^,^35^. It was suggested that the α_2_βγ heterotetramer contains two binding sites for each ligand, including Mn^2+^, ICT, NAD and ADP, which are probably located at the interfaces of the α and β subunits and the α and γ subunits^28^,^29^,^36^,^37^,^38^,^39^. In our previous work, we studied systematically the enzymatic properties of the α_2_βγ heterotetramer and the αβ and αγ heterodimers of human NAD-IDH (also called IDH3) and the specific functions of the β and γ subunits in the α_2_βγ heterotetramer^40^. Our biochemical data demonstrate that the αγ heterodimer exhibits similar enzymatic properties as the α_2_βγ heterotetramer and can be positively regulated by CIT and ADP, whereas the αβ heterodimer has only basal activity and cannot be regulated. Furthermore, we show that in the α_2_βγ heterotetramer, the γ subunit plays the regulatory role to activate the holoenzyme and the β subunit plays the structural role to facilitate the assembly and ensure the full activity of the holoenzyme^40^. However, the molecular basis for the assembly of the α_2_βγ heterotetramer and the molecular mechanism of the allosteric regulation of the αγ heterodimer and the α_2_βγ heterotetramer are still elusive.

In this work, to investigate how the γ subunit plays the regulatory role to activate the αγ heterodimer, we determined the crystal structures of the αγ heterodimer with the γ subunit bound without or with the positive regulators CIT and ADP, and carried out detailed mutagenesis and kinetic studies to validate the functional roles of the key residues involved in the binding of the regulators and the conformational changes. We found that the CIT binding induces significant conformational changes at the allosteric site, which are transmitted to the active site through the heterodimer interface, leading to stabilization of the ICT binding at the active site and thus activation of the enzyme. The ADP binding does not induce further conformational changes but enhances the CIT binding through Mg^2+^-mediated interactions, yielding a synergistic activation effect. Intriguingly, we also found that ICT can bind to the CIT-binding subsite, which induces similar conformational changes but exhibits a weaker activation effect. Our structural and biochemical data together reveal the molecular basis for the interplay of different regulators and the molecular mechanism of the allosteric regulation of the αγ heterodimer.

## Results

### Overall structure of the αγ heterodimer

The preparation and biochemical characterization of the αγ heterodimer of human NAD-IDH were described in details previously^40^. The crystal structures of the αγ heterodimer with the γ subunit bound without any regulators (α^Mg^γ), and bound with a Mg^2+^ and a CIT (α^Mg^γ^Mg+CIT^), with a Mg^2+^, a CIT and an ADP (α^Mg^γ^Mg+CIT+ADP^), and with a Mg^2+^, an ICT and an ADP (α^Mg^γ^Mg+ICT+ADP^), and the αγ_K151A_ mutant bound with a Mg^2+^, a CIT and an ADP (αγ_K151A_^Mg+CIT+ADP^) were determined at 2.8 Å, 2.3 Å, 2.65 Å, 2.8 Å, and 2.5 Å resolution, respectively (Table 1). These structures all belong to space group *P*3_1_21 with each asymmetric unit containing one αγ heterodimer. In all of these structures, the polypeptide chains of both the α and γ subunits are well defined with high-quality electron density except for a few N-terminal and/or C-terminal residues. At the active site or/and the allosteric site, there was strong electron density which was interpreted as Mg^2+^ due to the presence of Mg^2+^ (>0.2 mM) in the crystallization solutions (Supplementary Figure S1). In the ligand-bound structures, there was evident electron density at the allosteric site matching the corresponding ligand(s) very well (Supplementary Figure S1). The αγ heterodimer in different ligand-bound structures shows very similar overall structure with RMSD of <1.2 Å for over 650 Cα atoms when compared pair-wisely (Supplementary Table S1). However, there were notable conformational differences at the allosteric site, the active site and the heterodimer interface upon the binding of CIT (see discussion later).

**Figure 1:**
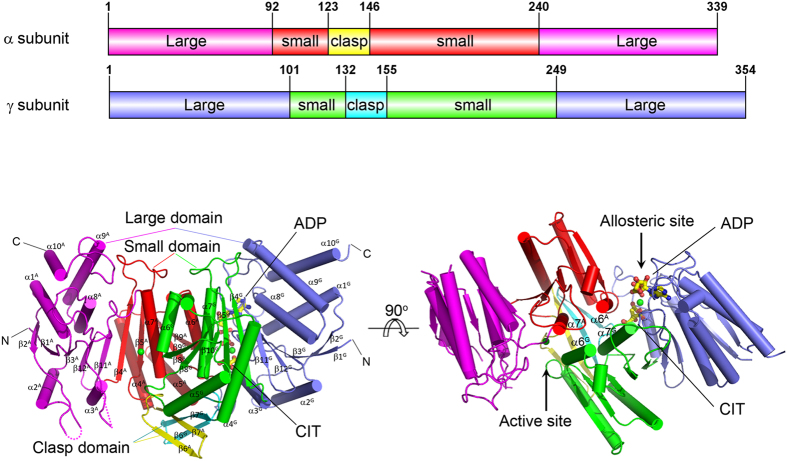
Structure of the αγ heterodimer of human NAD-IDH. Overall structure of the α^Mg^γ^Mg+CIT+ADP^ heterodimer in two different orientations. Left panel: viewing in perpendicular to the pseudo 2-fold axis of the αγ heterodimer. Right panel: viewing along the pseudo 2-fold axis of the αγ heterodimer. The color-coding schemes of individual domains of the α and γ subunits are shown above. The bound CIT and ADP in the γ subunit are shown as ball-and-stick models, and the bound Mg^2+^ ions as green spheres. Secondary structure elements of the α and γ subunits are labeled with superscripts “A” and “G”, respectively.

Similar to NADP-IDHs and yeast NAD-IDH, both the α and γ subunits consist of 10 α-helices (α1–α10) and 12 β-strands (β1–β12) which fold into three domains: a large domain assuming a typical Rossmann fold, a small domain assuming an α/β sandwich fold, and a clasp domain consisting of two anti-parallel β-strands (Fig. 1 and Supplementary Figure S2). The α and γ subunits form a compact heterodimer with a pseudo two-fold symmetry and the heterodimer interface is mainly mediated by extensive hydrophilic and hydrophobic interactions between the α6 and α7 helices of the small domains which form a four-helix bundle, and between the β6 and β7 strands of the clasp domains which form a four-stranded β-sheet (Fig. 1a). The active site is located in the cleft formed by the large and small domains of the α subunit and the small domain of the γ subunit, and comprises of the binding sites for the metal ion, substrate ICT and coenzyme NAD. The allosteric site is located in the cleft formed by the large and small domains of the γ subunit and the small domain of the α subunit, and comprises of the binding sites for the metal ion and activators CIT and ADP.

It is noteworthy that in all of these structures, two αγ heterodimers related by the crystallographic two-fold axis form a dimer of heterodimer or a heterotetramer via the four-stranded β-sheets of the clasp domains, which form an eight-stranded β-barrel. This is in agreement with our previous biochemical data showing that the αγ protein exists mainly as a heterodimer at low concentration in solution but as a dimer of heterodimer at high concentration^40^. As the dimer of the αγ heterodimer is formed in a similar manner as the IDH1/IDH2 heterotetramer of yeast NAD-IDH^21^, it is possible that the αγ heterotetramer might mimic the α_2_βγ heterotetramer of human NAD-IDH.

### Binding of CIT induces conformational changes at the allosteric site

**Figure 2:**
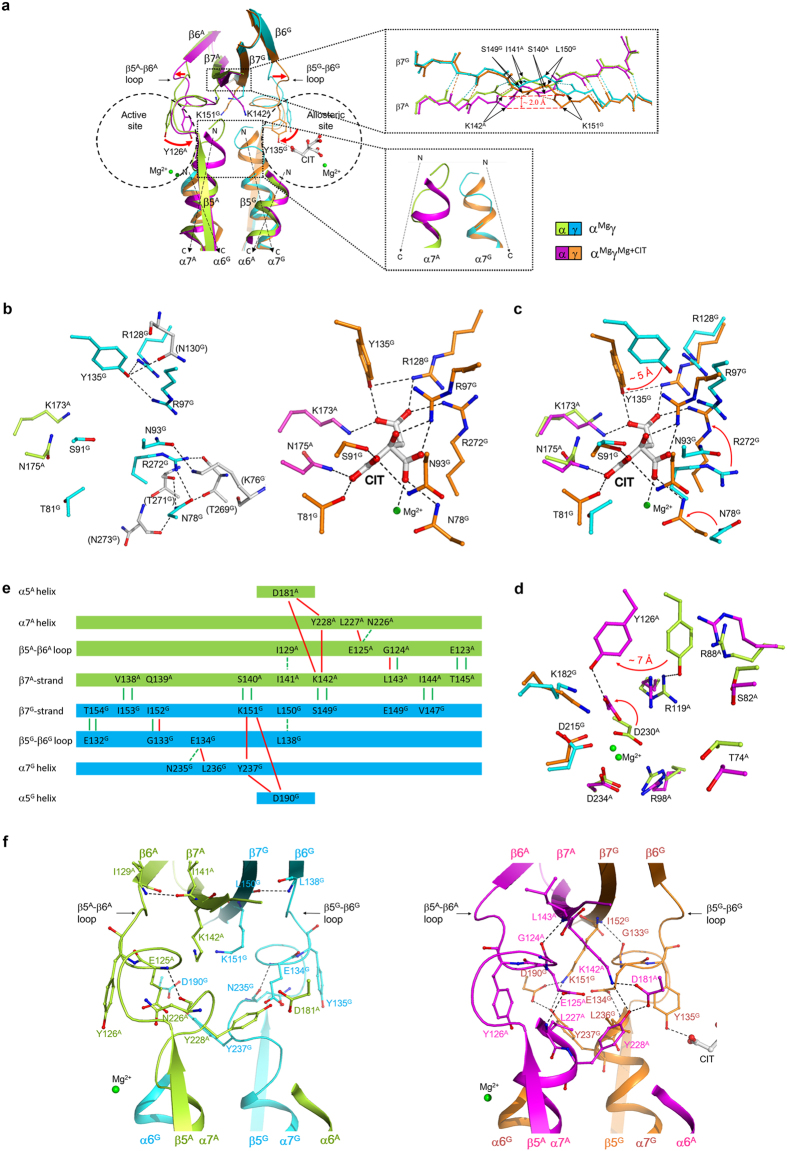
Binding of CIT induces conformational changes at the allosteric site, the active site, and the heterodimer interface. (**a**) Comparison of the α^Mg^γ and α^Mg^γ^Mg+CIT^ structures. The α and γ subunits in the α^Mg^γ structure are colored in lemon and cyan, respectively, and these in the α^Mg^γ^Mg+CIT^ structure in magenta and orange, respectively. Major conformational changes are observed at the allosteric site, the active site, and the β5–β6 loop, the α7 helix and the β7 strand at the heterodimer interface. The orientations of the α6 and α7 helices in both subunits are indicated with dashed arrows. Some key residues involved in the conformational changes are shown with side chains. The zoom-in panel on the right top shows the conformational changes of the β7^A^ and β7^G^ strands. For clarity, only the hydrogen-bonding interactions between the main chains are shown and the side chains are omitted. The zoom-in panel in the right bottom shows the loop-to-helix transition of the N-terminal region of the α7^A^ and α7^G^ helices. (**b**) Structure of the allosteric site in the α^Mg^γ (left panel) and α^Mg^γ^Mg+CIT^ (right panel) structures. In the α^Mg^γ structure, the hydrogen-bonding interactions between the residues of the CIT-binding subsite and the surrounding residues (colored in grey) are indicated with dashed lines. In the α^Mg^γ^Mg+CIT^ structure, the interactions between CIT and the surrounding residues and the Mg^2+^ are indicated with dashed lines. (**c**) Comparison of the allosteric site in the α^Mg^γ and α^Mg^γ^Mg+CIT^ structures. (**d**) Comparison of the active site in the α^Mg^γ and α^Mg^γ^Mg+CIT^ structures. (**e**) A schematic diagram showing the hydrogen-bonding interactions among the β5–β6 loop, the α7 helix, the β7 strand, and the α5 helix in the α^Mg^γ and α^Mg^γ^Mg+CIT^ structures. The interactions in the α^Mg^γ structure are indicated with green lines; these disrupted upon the CIT binding are indicated with dashed green lines and the newly formed interactions with red lines. (**f**) Structure of the heterodimer interface in the α^Mg^γ (left panel) and α^Mg^γ^Mg+CIT^ (right panel) structures. For clarity, only the hydrogen-bonding interactions altered upon the CIT binding are indicated with dashed lines.

The previous biochemical studies showed that mammalian NAD-IDHs can be activated by CIT through decreasing the *S*_*0.5*,ICT_ value^32^. Our biochemical data have shown that the αγ heterodimer can be activated by CIT in a manner similar to the α_2_βγ heterotetramer^40^. To investigate the regulatory mechanism of CIT activation, we determined the α^Mg^γ and α^Mg^γ^Mg+CIT^ structures (Table 1). Comparison of the two structures shows that the CIT binding does not cause notable conformational change in the overall structure (Supplementary Table S1), but induces significant conformational changes at the allosteric site, the active site, and the heterodimer interface (Fig. 2a).

In the α^Mg^γ structure, there are no metal ion and ligand bound at the allosteric site and the key residues composing the allosteric site are stabilized by several hydrogen bonds (Fig. 2b, left panel). Specifically, the side chain of Tyr135^G^ (residues of the α and γ subunits are superscripted by “A” and “G”, respectively) forms hydrogen bonds with the side chains of Arg97^G^, Arg128^G^ and Asn130^G^; the side chain of Asn93^G^ is oriented outwards from the CIT-binding subsite to form a hydrogen bond with the side chain of Lys76^G^; the side chain of Arg272^G^ points away from the CIT-binding subsite to interact with the side chain of Asn78^G^ and the main-chain carbonyl of Thr269^G^; and the side chain of Asn78^G^ also points away from the CIT-binding subsite to form hydrogen bonds with the side chains of Thr269^G^ and Arg272^G^ and the main-chain carbonyls of Thr271^G^ and Asn273^G^.

In the α^Mg^γ^Mg+CIT^ structure, there are a Mg^2+^ and a CIT bound at the allosteric site, and the CIT has extensive hydrogen-bonding interactions with the surrounding residues and forms a coordination bond with the Mg^2+^ (Fig. 2b, right panel). Specifically, the α-carboxyl group of CIT forms hydrogen bonds with the side chains of Asn175^A^, Thr81^G^, Ser91^G^ and Asn93^G^; the β-carboxyl group forms hydrogen bonds with the side chains of Asn78^G^ and Arg97^G^ and a coordination bond with the Mg^2+^; the γ-carboxyl group forms hydrogen bonds with the side chains of Lys173^A^, Arg128^G^, Tyr135^G^ and Arg272^G^; and the β-hydroxyl group forms a hydrogen bond with the side chain of Arg97^G^. The Mg^2+^ binds next to CIT and is coordinated by six ligands with an octahedral geometry, including the side chain of Asn78^G^, the main-chain carbonyl of Arg272^G^, the β-carboxyl of CIT, and three water molecules (Supplementary Figure S1c).

Comparison of the α^Mg^γ and α^Mg^γ^Mg+CIT^ structures shows that the CIT binding induces significant conformational changes of several key residues at the allosteric site to interact with the CIT, including Asn78^G^, Asn93^G^, Arg97^G^, Arg128^G^, Tyr135^G^ and Arg272^G^ (Fig. 2c). Upon the CIT binding, the hydrogen-bonding interactions between the side chains of Arg97^G^ and Tyr135^G^ and between the side chains of Arg272^G^ and Asn78^G^ in the α^Mg^γ structure are disrupted. Meanwhile, the side chain of Tyr135^G^ is rotated by about 30° with the hydroxyl group moving about 5 Å towards the CIT-binding subsite to form hydrogen bonds with the γ-carboxyl group of CIT and the side chain of Arg128^G^; the side chains of both Arg97^G^ and Arg128^G^ assume extended conformations to interact with the β-hydroxyl and β-carboxyl groups and the γ-carboxyl group of CIT, respectively; the side chains of Asn78^G^, Asn93^G^ and Arg272^G^ are also rotated towards the CIT-binding subsite to make hydrogen-bonding interactions with the β-carboxyl, the α-carboxyl and the γ-carboxyl groups of CIT, respectively.

It is noteworthy that in the α^Mg^γ structure, there is a Mg^2+^ bound at the active site but no Mg^2+^ bound at the allosteric site; and in the α^Mg^γ^Mg+CIT^ structure, there is a Mg^2+^ bound at both the active site and the allosteric site. This is the first time to identify a metal ion bound at the allosteric site. These results suggest that the binding of Mg^2+^ at the allosteric site is likely dependent on the binding of CIT, consistent with the observation that the CIT binding induces the conformational changes of Asn78^G^ and Arg272^G^, both of which make coordination bonds with the Mg^2+^ and form part of the Mg^2+^-binding subsite (Supplementary Figure S1c).

### Binding of CIT also induces conformational changes at the active site

Our biochemical data have shown that similar to the α_2_βγ heterotetramer, the αγ heterodimer requires a divalent metal ion for the enzymatic activity^40^. In both the α^Mg^γ and α^Mg^γ^Mg+CIT^ structures, there is a Mg^2+^ bound at the active site, which is coordinated by Asp230^A^, Asp234^A^, Asp215^G^ and water molecules (Supplementary Figure S1a,b). The functional roles of Asp230^A^, Asp234^A^ and Asp215^G^ of human NAD-IDH have been validated by the previous mutagenesis and biochemical data showing that mutations of these residues would substantially increase the *S*_*0.5*_ values for Mn^2+^ and/or ICT, and thus decrease the activity^37^. Sequence comparison shows that Asp230^A^ and Asp234^A^ of human NAD-IDH are strictly conserved in NADP-IDHs and the catalytic subunit of other NAD-IDHs, but are replaced by Asn or Thr in the regulatory subunits of NAD-IDHs; and Asp215^G^ is strictly conserved in both the catalytic and regulatory subunits of NAD-IDHs and in NADP-IDHs (Supplementary Figure S2). The corresponding residues in NADP-IDHs are also involved in the binding of the metal ion and some of them in the binding of ICT, and thus play vital roles in the catalytic reaction^13^,^14^,^15^.

Sequence comparison of human NAD-IDH with NADP-IDHs and other NAD-IDHs shows that the key residues involved in the binding of ICT at the active site are strictly conserved, including Thr74^A^, Ser82^A^, Arg88^A^, Arg98^A^, Arg119^A^, Tyr126^A^, Asp230^A^, Lys182^G^ and Asp215^G^ (Supplementary Figure S2). Very intriguingly, comparison of the α^Mg^γ and α^Mg^γ^Mg+CIT^ structures shows that although most of the residues composing the active site exhibit no significant conformational differences, Tyr126^A^ and Asp230^A^ undergo marked conformational changes (Fig. 2d). In the α^Mg^γ structure, the side chain of Tyr126^A^ forms a hydrogen bond with the side chain of Arg119^A^ and the side chain of Asp230^A^ forms two coordination bonds with the Mg^2+^; whereas in the α^Mg^γ^Mg+CIT^ structure, the side chain of Tyr126^A^ is rotated by about 30° with the hydroxyl group moving about 7 Å to form a hydrogen bond with the side chain of Asp230^A^, and concurrently the side chain of Asp230^A^ is rotated towards Tyr126^A^ and additionally forms two coordination bonds with the Mg^2+^ via the side-chain carboxyl and main-chain carbonyl groups. Further structural comparison shows that the side-chain conformations of Tyr126^A^ and Asp230^A^ in the α^Mg^γ^Mg+CIT^ structure are similar to those in the ICT-bound PmIDH and HcIDH structures, in which the equivalent residues (Tyr140 and Asp275 of PmIDH and Tyr139 and Asp275 of HcIDH) form a hydrogen bond with each other and additionally make hydrogen-bonding interactions with ICT^14^,^15^ (Supplementary Figure S3). This implies that the side chains of Tyr126^A^ and Asp230^A^ in the α^Mg^γ^Mg+CIT^ structure are in proper conformations to bind ICT. These results indicate that the CIT binding at the allosteric site induces marked conformational changes of two important residues at the active site, which could facilitate the substrate binding. This is consistent with the biochemical data showing that the *S*_*0.5,ICT*_ of the αγ heterodimer is notably decreased in the presence of CIT^40^. Nevertheless, due to the absence of ICT, the side chains of Arg88^A^ and Arg98^A^ in the α^Mg^γ^Mg+CIT^ structure are not in optimal conformations to interact ICT as observed in the ICT-bound PmIDH and HcIDH structures (Supplementary Figure S3), suggesting that ICT binding would induce further conformational changes of the active site.

### Conformational changes at the allosteric site are transmitted to the active site via the heterodimer interface

To understand the molecular basis for the communication between the allosteric site and the active site, we carried out a detailed comparison of the α^Mg^γ and α^Mg^γ^Mg+CIT^ structures and found that upon the CIT binding, in addition to the conformational changes at the allosteric site and the active site, several structure elements at the heterodimer interface, especially the β5–β6 loop and the α7 helix of the small domain, and the β7 strand of the clasp domain in both the α and γ subunits, also undergo substantial conformational changes in a pseudo symmetric manner (Fig. 2a). In the absence of CIT (the α^Mg^γ structure), in both subunits, the N-terminal region of the α7 helix adopts a loop conformation and there are a number of hydrogen-bonding interactions among residues of these structure elements (Fig. 2e,f left panel). Specifically, in the γ-subunit, the side chain of Glu132^G^ forms a hydrogen bond each with the main-chain amine and the side chain of Thr154^G^; the main-chain amine of Gly133^G^ forms a hydrogen bond with the main-chain carbonyl of Ile152^G^; the main-chain amine of Glu134^G^ forms a hydrogen bond with the main-chain carbonyl of Asn235^G^; and the main-chain amine of Leu138^G^ forms a hydrogen bond with the main-chain carbonyl of Leu150^G^; however, there is no hydrogen-bonding interaction between the α7^G^ helix and the β7^G^ strand. Similarly, in the α-subunit, the side chain of Glu123^A^ forms a hydrogen bond each with the main-chain amine and the side chain of Thr145^A^; the main-chain amine of Gly124^A^ forms a hydrogen bond with the main-chain carbonyl of Leu143^A^; the main-chain amine of Glu125^A^ forms a hydrogen bond with the main-chain carbonyl of Asn226^A^; and the main-chain amine of Ile129^A^ forms a hydrogen bond with the main-chain carbonyl of Ile141^A^; but there is no hydrogen-bonding interaction between the α7^A^ helix and the β7^A^ strand.

Upon the CIT binding (the α^Mg^γ^Mg+CIT^ structure), in both subunits, the C-terminal region of the β5–β6 loop adopts an alternative conformation; the N-terminal region of the α7 helix assumes an α-helical conformation to form a long α7 helix; and the β7 strand bends towards the β5–β6 loop and the α7 helix (the Cα atoms of Ser149^G^-Lys151^G^ and Ser140^A^-Lys142^A^ are shifted by about 1–2 Å) (Fig. 2a). Consequently, some of the hydrogen-bonding interactions among these structure elements in the α^Mg^γ structure are disrupted, and a new and more intensive network of hydrogen-bonding interactions is established (Fig. 2e,f right panel). Specifically, in the γ-subunit, accompanying the conformational changes of Arg97^G^ and Tyr135^G^, the hydrogen bonds between Glu134^G^ and Asn235^G^ and between Leu138^G^ and Leu150^G^ are disrupted. On the other hand, the main chain of Gly133^G^ forms a new hydrogen bond with the main chain of Ile152^G^; the side chain of Glu134^G^ is inserted into the space formerly occupied by the N-terminal loop of the α7^G^ helix and forms a new hydrogen bond with the main-chain amine of Leu236^G^; the side chain of Lys151^G^ forms two hydrogen bonds with the side chains of Tyr237^G^ and Asp190^G^, and the side chains of Tyr237^G^ and Asp190^G^ also form a hydrogen bond with each other. Similarly, in the α subunit, the hydrogen bonds between Glu125^A^ and Asn226^A^ and between Ile129^A^ and Ile141^A^ are disrupted. Meanwhile, the main chain of Gly124^A^ gains a hydrogen bond with the main chain of Leu143^A^; the side chain of Glu125^A^ is inserted into the space formerly occupied by the N-terminal loop of the α7^A^ helix and forms a hydrogen bond with the main-chain amine of Leu227^A^; the side chain of Lys142^A^ forms hydrogen bonds with the side chains of Tyr228^A^ and Asp181^A^, and the side chains of Tyr228^A^ and Asp181^A^ form a hydrogen bond with each other. Consequently, the side chain of Tyr126^A^ is rotated towards the active site and assumes a proper position to interact with ICT. The conformational changes of these structure elements at the heterodimer interface and the alterations of the hydrogen-bonding interactions among the associated residues upon the CIT binding provide the molecular basis for the transmission of the conformational changes from the allosteric site to the active site.

### Binding of ADP induces no further conformational changes but enhances the CIT binding at the allosteric site

**Figure 3:**
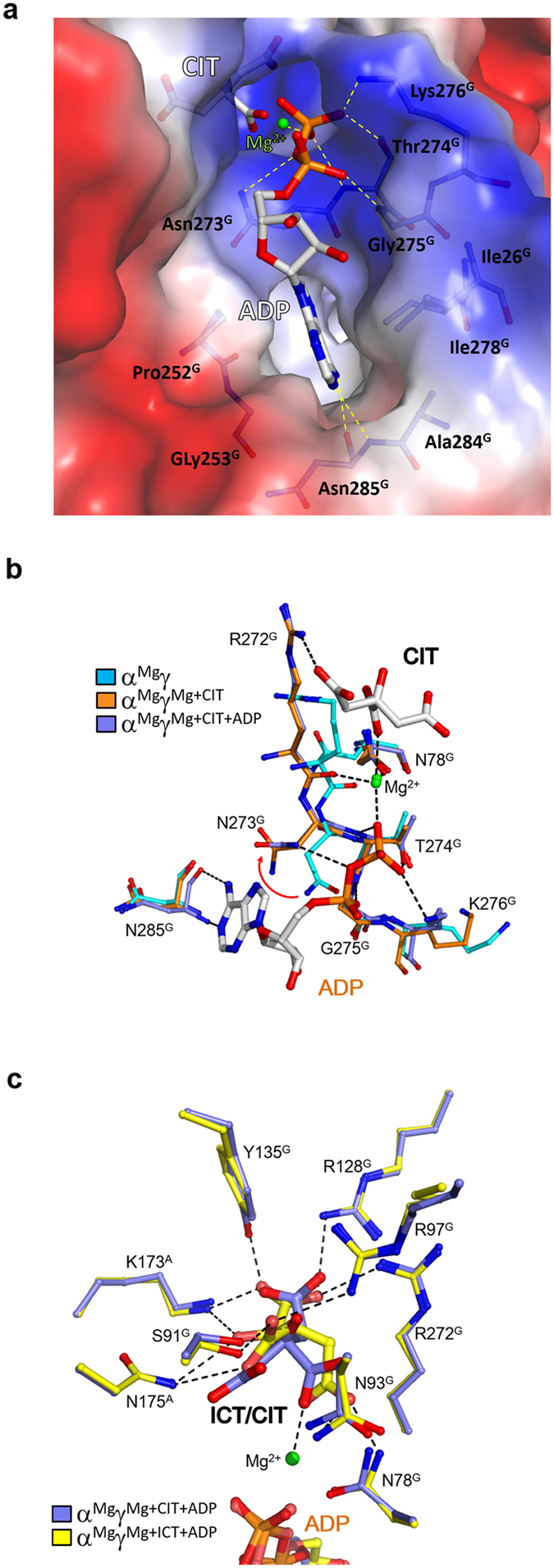
Binding of ADP induces no further conformational changes. (**a**) Structure of the ADP-binding subsite in the α^Mg^γ^Mg+CIT+ADP^ structure. The protein is shown with electrostatic potential surface, the bound CIT and ADP are shown with ball-and-stick models, the Mg^2+^ with a green sphere, and the surrounding residues with side chains. The hydrophilic interactions of ADP with the surrounding residues and the Mg^2+^ are indicated with dashed lines. (**b**) Comparison of the ADP-binding subsite in the α^Mg^γ (cyan), α^Mg^γ^Mg+CIT^ (orange) and α^Mg^γ^Mg+CIT+ADP^ (slate) structures. In the α^Mg^γ^Mg+CIT^ and α^Mg^γ^Mg+CIT+ADP^ structures, the side chain of Asn273^G^ rotates about 100° away from the ADP-binding subsite compared to that in the α^Mg^γ structure. (**c**) Comparison of the CIT-binding subsite in the α^Mg^γ^Mg+ICT+ADP^ (yellow) and α^Mg^γ^Mg+CIT+ADP^ (slate) structures. The bound ICT, CIT and ADP are shown with ball-and-stick models, the Mg^2+^ with a green sphere, and the surrounding residues with side chains. The hydrophilic interactions of ICT with the surrounding residues and the Mg^2+^ are indicated with dashed lines. ICT binds to the CIT-binding subsite and induces similar conformational changes.

The previous biochemical studies showed that mammalian NAD-IDHs can be activated by ADP through decreasing the *S*_*0.5*,ICT_ as well^35^,^36^. Our biochemical data have also shown that the αγ heterodimer and the α_2_βγ heterotetramer can be activated by ADP, and additionally the activation effect of CIT and ADP together is more dramatic than CIT or ADP alone, indicating that the two activators work synergistically^40^. To investigate the molecular mechanisms of the ADP activation and the synergistic effect of CIT and ADP, we sought but failed to obtain crystals of the αγ heterodimer bound with ADP alone at the allosteric site using either co-crystallization or soaking methods; however, in the presence of CIT and ADP, we obtained crystals and thus determined the structure of the α^Mg^γ^Mg+CIT+ADP^ heterodimer. In this structure, ADP binds next to CIT and Mg^2+^ at the allosteric site and has both hydrophobic and hydrophilic interactions with several residues of the γ subunit (Fig. 3a). Specifically, the adenine moiety of ADP binds to a hydrophobic pocket composed of Ile26^G^, Pro252^G^, Ile278^G^, Gly253^G^ and Ala284^G^, and additionally forms two hydrogen bonds with the main-chain amine and carbonyl of Asn285^G^ at the deep end of the pocket. The α-phosphate of ADP forms hydrogen bonds with the side chain of Asn273^G^ and the main-chain amine of Gly275^G^; and the β-phosphate forms hydrogen bonds with the side chains of Lys276^G^ and Thr274^G^ and the main-chain amine of Thr274^G^, and additionally makes a coordination bond with the Mg^2+^.

Intriguingly, comparison of the α^Mg^γ^Mg+CIT^ and α^Mg^γ^Mg+CIT+ADP^ structures shows that the key residues composing the allosteric site, the active site and the structure elements at the heterodimer interface assume almost identical conformations in both structures, indicating that the binding of ADP does not induce further conformational changes (Supplementary Figure S4). On the other hand, comparison of the α^Mg^γ^Mg+CIT^ and α^Mg^γ^Mg+CIT+ADP^ structures with the α^Mg^γ structure shows that upon the CIT binding, several residues in the β12^G^–α8^G^ loop (residues 272^G^–276^G^) at the allosteric site undergo notable conformational changes to assume positions that are suitable not only for the CIT and Mg^2+^ binding but also for the ADP binding (Fig. 3b). Particularly, in the α^Mg^γ structure, the side chain of Asn273^G^ partially occupies the space for the α,β-phosphates of ADP and forms a hydrogen bond with the main-chain amine of Gly275^G^; whereas in the α^Mg^γ^Mg+CIT^ and α^Mg^γ^Mg+CIT+ADP^ structures, the side chain of Asn273^G^ is rotated away by about 100° and thus releases the phosphate-binding site. Our crystallization results and structural data together indicate that ADP cannot stably bind to the allosteric site by itself, and the binding of CIT (and Mg^2+^) induces proper conformational changes of the allosteric site to facilitate or stabilize the binding of ADP and hence is likely to precede the ADP binding. On the other hand, the structural analysis shows that the binding of ADP establishes a more extensive network of hydrophilic and hydrophobic interactions among CIT, ADP and the surrounding residues mediated by the Mg^2+^, which conversely enhances or stabilizes the CIT and Mg^2+^ binding. This interplay of CIT and ADP provides the molecular basis for the synergistic activation effect of the two activators.

### ICT can bind to the CIT-binding subsite and induces similar conformational changes

Our kinetic data showed that the αγ heterodimer exhibits a Hill coefficient of 2 for ICT in the absence of CIT but a Hill coefficient of 1 in the presence of CIT, indicating that the αγ heterodimer has two cooperative ICT-binding sites, one of which is blocked or occupied by CIT upon the CIT binding^40^. In the absence of ADP, we failed to obtain crystals of the αγ heterodimer bound with ICT at the allosteric site using either co-crystallization or soaking methods. However, in the presence of both ICT and ADP, we obtained crystals and thus determined the structure of the α^Mg^γ^Mg+ICT+ADP^ heterodimer. In this structure, ICT binds to the CIT-binding subsite with a similar orientation as CIT and forms very similar hydrogen-bonding interactions with the surrounding residues (Fig. 3c). Specifically, the α-carboxyl group of ICT makes hydrogen-bonding interactions with the side chains of Lys173^A^, Arg128^G^, Tyr135^G^, and Arg272^G^; the α-hydroxyl group makes hydrogen-bonding interactions with the side chains of Lys173^A^ and Asn175^A^; the β-carboxyl group makes hydrogen-bonding interactions with the side chains of Asn175^A^, Ser91^G^ and Arg97^G^; and the γ-carboxyl group forms hydrogen-bonding interactions with the side chains of Asn78^G^ and Asn93^G^ and makes a coordination bond with the Mg^2+^. Moreover, the residues composing the allosteric site, the active site and the structure elements at the heterodimer interface assume almost identical conformations as those in the α^Mg^γ^Mg+CIT+ADP^ structure (Supplementary Figure S4). The crystallization results and structural data together indicate that like ADP, ICT cannot stably bind to the allosteric site alone, but can bind to the CIT-binding subsite in the presence of ADP which induces similar conformational changes as the binding of CIT and ADP. These results also imply that the binding of ICT (and ADP) should have an activation effect which is however weaker than the binding of CIT (and ADP). Intriguingly, our previous biochemical data showed that compared to that in the absence of any activators, the *S*_*0.5*,ICT_ of the αγ heterodimer is decreased by 1.7, 2.7, and 24.7 folds in the presence of CIT, ADP and both activators, respectively^40^, suggesting that ADP has a slightly stronger activation effect than CIT, which seems to be in contradiction with the structural data showing that ADP cannot bind to the allosteric site alone but can bind to the allosteric site in the presence of CIT or ICT. This discrepancy can now be explained very well: because both ADP and ICT exist in the kinetic assay, the apparent activation effect of ADP is in fact the combined activation effect of ADP and ICT, which is slightly stronger than that of CIT alone but is much weaker than that of CIT and ADP. Moreover, our structural data suggest that the apparent *S*_*0.5*,ICT_ of the αγ heterodimer in the absence of any activators contains the contribution of the weak activation of ICT.

### Biochemical studies of the functional roles of the key residues

Our structural data show that the CIT binding induces conformational changes of a number of conserved residues at the allosteric site, the active site and the heterodimer interface, and the ADP binding does not induce further conformational changes but enhances the CIT binding via formation of a more extensive network of hydrogen-bonding interactions mediated by the metal ion. To validate the functional roles of these residues, we performed mutagenesis and kinetic studies to analyze their effects on the allosteric activation of the αγ heterodimer (Table 2).

Firstly, we analyzed the functional roles of the residues of the allosteric site involved in the CIT binding in the absence and presence of CIT (Table 2). In the absence of CIT, mutations of most of these residues slightly increase the *S*_*0.5*,ICT_ by 1–2 folds and moderately decrease the catalytic efficiency (*k*cat*/ S*_*0.5*,ICT_) by 3–10 folds compared to the wild-type enzyme. Mutation γ-R272A has a more severe effect on the *S*_*0.5*,ICT_ (increased by 4.3 folds) and the catalytic efficiency (decreased by 10.0 folds). Mutations γ-N78A and γ-S91A are exceptions which have less critical effects on both the *S*_*0.5*,ICT_ and the catalytic efficiency. These results support the notion that the binding of ICT alone has a weak activation effect, and mutations of most of the residues involved in the CIT binding at the allosteric site diminish the weak activation effect of ICT.

In the presence of CIT, the *S*_*0.5*,ICT_ of the wild-type enzyme is decreased by 7.6 folds and the catalytic efficiency is increased by 12.9 folds, indicating a strong activation effect of CIT. In contrast, compared to these in the absence of CIT, the *S*_*0.5*,ICT_ of most of the mutants is only decreased by 1–2 folds and the catalytic efficiency is only increased by 1–2 folds, indicating that mutations of these residues significantly impair the CIT activation effect (Table 2). Again, mutations γ-N78A and γ-S91A are exceptions which have less critical impacts on the CIT activation: mutation γ-N78A causes a 9.5-fold decrease in the *S*_*0.5*,ICT_ and a 15.1-fold increase in the catalytic efficiency; and mutation γ-S91A causes a 4.0-fold decrease in the *S*_*0.5*,ICT_ and a 8.9-fold increase in the catalytic efficiency. These results suggest that most of the residues involved in the CIT binding play an important role and Asn78^G^ and Ser91^G^ a less essential role in the binding of CIT.

Secondly, we analyzed the functional roles of the residues of the allosteric site involved in the ADP binding in the absence and presence of ADP (Table 2). In the absence of ADP, mutations of these residues have less critical effects on the *S*_*0.5*,ICT_ (increased by about 1.5 folds) and the catalytic efficiency (decreased by <2 folds), except for γ-N285A which severely impairs the enzymatic activity. In the presence of ADP, the wild-type enzyme exhibits a 2.7-fold lower *S*_*0.5*,ICT_ and a 3.4-fold higher catalytic efficiency, indicating a moderate activation effect. Mutations γ-N273A and γ-T274A substantially compromise the activation effect of ADP, which cause only <1.2-fold decrease in the *S*_*0.5*,ICT_ and <1.2-fold increase in the catalytic efficiency compared to these in the absence of ADP; however, mutation γ-K276A has a less critical effect on the ADP activation, which causes a 3.6-fold decrease in the *S*_*0.5*,ICT_ and a 5.4-fold increase in the catalytic efficiency. As an exception, mutation γ-N285A completely abolishes the activity in the presence of ADP. These results suggest that Asn285^G^ plays a critical role, Asn273^G^ and Thr274^G^ play an important role, and Lys276^G^ play a less essential role in the binding of ADP.

Thirdly, we analyzed the functional roles of the key residues at the heterodimer interface involved in the structural communication between the allosteric site and the active site in the absence and presence of CIT and ADP (Table 2). In the absence of the activators, mutations γ-E134A and α-E125A have insignificant effects on the *S*_*0.5*,ICT_ (increased by <1.1 folds) and the catalytic efficiency (decreased by <2.1 folds); mutation α-D181A completely abolishes the activity; and the other mutations have moderate to severe effects on the *S*_*0.5*,ICT_ (increased by 1.2–3.3 folds) and the catalytic efficiency (decreased by 5.0–47.0 folds). In the presence of CIT and ADP, the wild-type enzyme is significantly activated with the *S*_*0.5*,ICT_ being decreased by 25.0 folds and the catalytic efficiency being increased by 44.5 folds. Again, mutations γ-E134A and α-E125A have no significant impact on the activation and these two mutants exhibit comparable *S*_*0.5*,ICT_ and catalytic efficiency as the wild-type enzyme; mutation α-D181A completely abolishes the activity; and the other mutations severely impair or completely abolish the activation effect and these mutants exhibit a slightly decreased or increased *S*_*0.5*,ICT_ (−2.4 to +1.2 folds) and catalytic efficiency (−1.2 to +4.5 folds) compared to these in the absence of the activators. These results suggest that Lys151^G^, Asp190^G^, Tyr237^G^, Lys142^A^, Asp181^A^, and Tyr228^A^ play an important role but Glu134^G^ and Glu125^A^ a less critical role in the transmission of the conformational changes from the allosteric site to the active site upon the binding of CIT and ADP.

Analyses of the kinetic data also show that the wild-type αγ heterodimer exhibits a Hill coefficient of 2 for ICT in the absence of CIT but a Hill coefficient of 1 in the presence of CIT or CIT and ADP (Table 2), indicating that the αγ heterodimer has two cooperative ICT-binding sites, one of which is occupied by CIT upon the CIT binding. Consistent with their effects on the *S*_*0.5*,ICT_ and the catalytic efficiency, mutations of the key residues involved in the CIT binding except for the γ-N78A and γ-S91A mutations abolish the cooperativity in the absence of CIT because these mutations impair the binding of ICT at the allosteric site; and mutations of the key residues at the heterodimer interface except for the γ-E134A and α-E125A mutations also abolish the cooperativity in the absence of CIT and ADP because these mutations disrupt the structural communication between the allosteric site and the active site. Like the wild-type enzyme, all the mutants suppress the cooperativity in the presence of CIT or CIT and ADP. The exceptions are the γ-N78A, γ-S91A, γ-E134A and α-E125A mutants which exhibit a Hill coefficient of about 1.7 for ICT in the absence of CIT (and ADP), indicating the existence of two cooperative ICT-binding sites (Table 2). This is in agreement with the biochemical data showing that these mutations have no significant effects on the CIT binding and the CIT activation. On the other hand, the wild-type αγ heterodimer exhibits a Hill coefficient of 2 for ICT in the absence of ADP and a Hill coefficient of 1.6 in the presence of ADP (Table 2), indicating that the αγ heterodimer still has two ICT-binding sites with positive cooperativity in the presence of ADP. Furthermore, mutations of the residues involved in the ADP binding do not abolish the cooperativity (with the Hill coefficient of >1.5) in both the absence and presence of ADP. These results are consistent with the structural data showing that the allosteric site can bind ICT in the presence of ADP, and mutations of the residues involved in the ADP binding do not disrupt the binding of CIT (or ICT) at the allosteric site.

### Mutation γ-K151A disrupts the structural communication between the allosteric site and the active site

**Figure 4:**
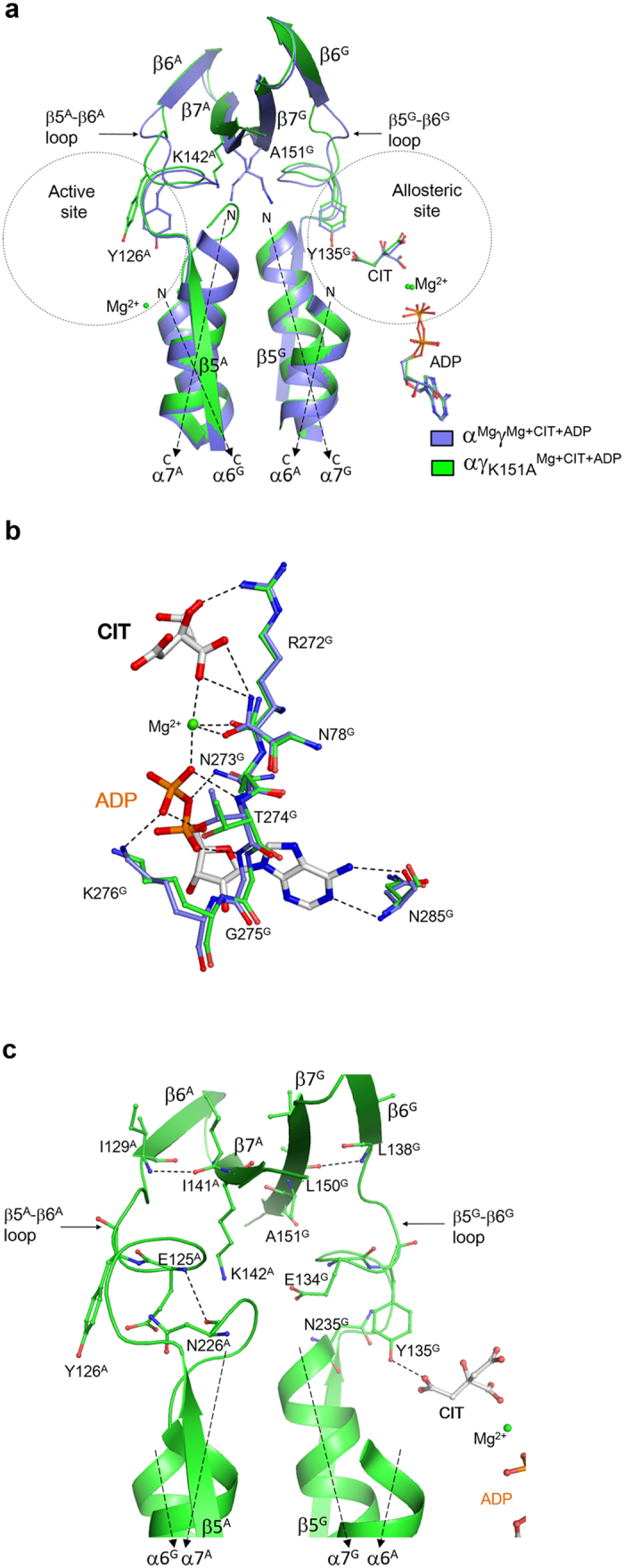
Mutation γ-K151A disrupts the structural communication between the allosteric site and the active site. (**a**) Comparison of the α^Mg^γ^Mg+CIT+ADP^ (slate) and αγ_K151A_^Mg+CIT+ADP^ (green) structures. No major conformational changes are found at the allosteric site. However, significant conformational differences are observed in the C-terminal region of the β5–β6 loop and the β7 strand of both the α and γ subunits, the N-terminal region of the α7 helix of the α subunit, and the active site. (**b**) Comparison of the allosteric site in the α^Mg^γ^Mg+CIT+ADP^ (slate) and αγ_K151A_^Mg+CIT+ADP^ (green) structures. The residues involved in the binding of CIT, Mg^2+^ and ADP adopt almost identical conformations in these two structures. The hydrogen-bonding interactions in the α^Mg^γ^Mg+CIT+ADP^ structure are indicated with dashed lines. (**c**) Conformations of the structure elements at the heterodimer interface including the β5–β6 loop, the α7 helix and the β7 strand of both the α and γ subunits in the αγ_K151A_^Mg+CIT+ADP^ structure. The key residues involved in the structural communication between the allosteric site and the active site are shown with side chains, and the hydrogen-bonding interactions are indicated with dashed lines.

Our structural and biochemical data reveal that Lys151^G^, Asp190^G^ and Tyr237^G^ of the γ subunit, and Lys142^A^, Asp181^A^ and Tyr228^A^ of the α subunit at the heterodimer interface play critical roles in the transmission of the conformational changes from the allosteric site to the active site through the alteration of hydrogen-bonding interactions, and mutations of these residues abolish the activation effect of CIT and ADP (Fig. 2 and Table 2). To investigate the structural basis for the functional roles of these residues in the allosteric regulation, we took the γ-K151A mutation as a representative and determined the crystal structure of the αγ_K151A_ mutant bound with Mg^2+^, CIT and ADP at the allosteric site (Table 1). In the αγ_K151A_^Mg+CIT+ADP^ structure, the structure of the allosteric site is very similar to that in the α^Mg^γ^Mg+CIT+ADP^ structure with the CIT, ADP and Mg^2+^ binding to the allosteric site in similar manners and maintaining almost identical interactions (Fig. 4a,b and Supplementary Figure S4a,b). In addition, accompanying with the conformational changes of Arg97^G^ and Tyr135^G^, the N-terminal part of the β5^G^–β6^G^ loop and the N-terminal region of the α7^G^ helix at the heterodimer interface also undergo similar conformational changes as in the α^Mg^γ^Mg+CIT+ADP^ structure. However, the C-terminal part of the β5^G^–β6^G^ loop and the β7^G^ strand assume similar conformations as in the α^Mg^γ structure (Fig. 4a,c and Supplementary Figure S4a). Specifically, the hydrogen bond between the main-chain amine of Glu134^G^ and the main-chain carbonyl of Asn235^G^ is disrupted and the side chain of Glu134^G^ is inserted into the space formerly occupied by the N-terminal loop of the α7^G^ helix and forms a hydrogen bond with the main-chain amine of Leu236^G^. Due to the γ-K151A mutation, the hydrogen-bonding interactions of Lys151^G^ with Asp190^G^ and Tyr237^G^ are lost but the hydrogen bond between the main-chain amine of Leu138^G^ and the main-chain carbonyl of Leu150^G^ is maintained. As a result, the β7^G^ strand has no conformational change, and consequently the β7^A^ strand, the β5^A^–β6^A^ loop and the N-terminal region of the α7^A^ helix also have no conformational changes and adopt similar conformations as in the α^Mg^γ structure (Fig. 4a,c and Supplementary Figure S4a,c). These results demonstrate that although the αγ_K151A_ mutant can bind CIT and ADP and induce conformational changes of the allosteric site and some structural elements of the heterodimer interface in the γ subunit, the γ-K151A mutation disrupts the structural communication between the γ subunit and the α subunit and hence the conformational changes at the allosteric site cannot be transmitted to the active site.

## Discussion

In this work, we determined a series of structures of the αγ heterodimer bound without or with the activator(s) (CIT, ICT and ADP) at the allosteric site. Analyses of those structures reveal the conformational changes at the allosteric site, the active site, and the heterodimer interface upon the binding of the activator(s) and identify the key residues involved in the transmission of the conformational changes from the allosteric site to the active site. The functional roles of these residues are validated by mutagenesis and kinetic data. The structural and biochemical data together demonstrate that CIT can bind independently to the allosteric site and the CIT binding induces significant conformational changes at the allosteric site, which are transmitted to the active site via the conformational changes of several structure elements at the heterodimer interface, including the β5–β6 loop, the N-terminal region of the α7 helix, and the β7 strand in both the α and γ subunits. These conformational changes are accompanied with the alterations of hydrogen-bonding interactions, leading to the active site to adopt a proper conformation suitable for the substrate binding. In addition, the conformational changes at the allosteric site induced by the CIT binding lead to the formation of the binding subsite for ADP and thus facilitate the binding of ADP. Although the ADP binding does not induce further conformational changes at the allosteric site, it establishes a more extensive hydrogen-bonding network between CIT and ADP mediated by the metal ion and hence enhances or stabilizes the CIT binding. Therefore, the binding of CIT and ADP together has a synergistic activation effect. Furthermore, our structural and biochemical data demonstrate that ICT cannot stably bind to the CIT-binding subsite alone, but can bind in the presence of ADP which induces similar conformational changes as the CIT binding, indicating that the allosteric site has a lower binding affinity for ICT than for CIT and thus the binding of ICT (and ADP) would have a weaker activation effect than the binding of CIT (and ADP). These results suggest that the apparent *S*_*0.5*,ICT_ of the αγ heterodimer in the absence of any activators actually contains the contribution of the weak activation effect of ICT, and the apparent activation effect of ADP is in fact the combined effect of ICT and ADP, explaining why the activation effect of ADP alone is slightly higher than that of CIT alone but is much weaker than that of CIT and ADP.

**Figure 5:**
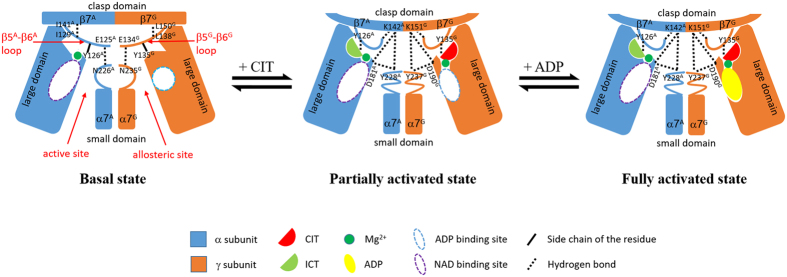
A schematic diagram showing the molecular mechanism of the allosteric regulation of the αγ heterodimer. In the absence of any activators, the active site adopts an inactive conformation unfavorable for the ICT binding, and the enzyme is in the basal state which has a high *S*_*0.5*,ICT_ with a low catalytic efficiency. The binding of CIT induces conformational changes at the allosteric site, which are transmitted to the active site through conformational changes of the structure elements at the heterodimer interface, including the β5–β6 loop, the α7 helix, and the β7-strand in both the α and γ subunits, leading to the conversion of the active site from the inactive conformation to the active conformation favorable for the ICT binding. Hence, the enzyme assumes the partially activated state which has a moderately decreased *S*_*0.5*,ICT_ with a moderately increased catalytic efficiency. The binding of ADP in the presence of CIT does not induce further conformational changes at the allosteric site and the active site, but establishes a more extensive hydrogen-bonding network among CIT, ADP and the surrounding residues through the metal ion, which conversely enhances or stabilizes the CIT binding. Hence, the binding of CIT and ADP together has a synergistic activation effect, and the enzyme assumes the fully activated state which has a substantially decreased *S*_*0.5*,ICT_ with a significantly increased catalytic efficiency.

Based on our structural and biochemical data, we can propose the molecular mechanism for the allosteric regulation of the αγ heterodimer of human NAD-IDH (Fig. 5). The α^Mg^γ structure represents the basal state of the enzyme. In this state, in both the α and γ subunits, the N-terminal region of the α7 helix adopts a loop conformation, and the β5–β6 loop interacts with the N-terminal region of the α7 helix and the β7 strand via several hydrogen bonds, but there is no direct interaction between the α7 helix and the β7 strand. Particularly, the side chain of Tyr126^A^ at the active site assumes a conformation unsuitable for the ICT binding, and therefore the basal state of the enzyme has a high S_0.5,ICT_ and hence a low catalytic efficiency.

The α^Mg^γ^Mg+CIT^ structure represents the partially activated state of the enzyme. In this state, the binding of CIT induces substantially conformational changes of several key residues (particularly Tyr135^G^) at the allosteric site, which further induce conformational change of the β5^G^–β6^G^ loop. Thus, several residues of the β5^G^–β6^G^ loop change their hydrogen-bond interactions with residues of the α7^G^ helix and the β7^G^ strand, which subsequently transduce the conformational changes of the α7^G^ helix and the β7^G^ strand at the heterodimer interface. Particularly, the hydrogen bond between the main-chain amine of Glu134^G^ (the β5^G^–β6^G^ loop) and the main-chain carbonyl of Asn235^G^ (the α7^G^ helix) is disrupted and the N-terminal region of the α7^G^ helix undergoes conformational change to transform from a loop conformation to an α-helical conformation. As a result, the side chain of Tyr237^G^ (the α7^G^ helix) is in a proper position to form a tripartite hydrogen-bonding network with the side chains of Asp190^G^ (the α5^G^ helix) and Lys151^G^ (the β7^G^ strand). Concurrently, the hydrogen bond between the main-chain amine of Leu138^G^ (the β5^G^–β6^G^ loop) and the main-chain carbonyl of Leu150^G^ (the β7^G^ strand) is also disrupted and a new hydrogen bond is formed between the main-chain carbonyl of Gly133^G^ (the β5^G^–β6^G^ loop) and the main-chain amine of Ile152^G^ (the β7^G^ strand). These two aspects together stabilize the interactions of the β5^G^–β6^G^ loop, the α7^G^ helix and the β7^G^ strand and thus induce and/or stabilize the bending of the β7^G^ strand around residues Ser149^G^-Lys151^G^.

The conformational changes of the allosteric site and the structure elements at the heterodimer interface in the γ subunit are then transmitted to the α subunit and the active site in a pseudo symmetric manner. In the αγ heterodimer, the β6 and β7 strands of the clasp domain of the α and γ subunits form a four-stranded anti-parallel β-sheet at the heterodimer interface, and the extensive hydrogen-bonding interactions between main chains of the residues of the β7^G^ and β7^A^ strands form the core of the β-sheet. The bending of the β7^G^ strand around residues Ser149^G^-Lys151^G^ upon the CIT binding induces the bending of the β7^A^ strand around residues Ser140^A^-Lys142^A^ towards the β5^A^-β6^A^ loop and the α7^A^ helix. This conformational change breaks up the hydrogen-bonding interaction between the main-chain carbonyl of Ile141^A^ (the β7^A^ strand) and the main-chain amine of Ile129^A^ (the β5^A^–β6^A^ loop) and forms a new hydrogen-bonding interaction between the main-chain amine of Leu143^A^ (the β7^A^ strand) and the main-chain carbonyl of Gly124^A^ (the β5^A^–β6^A^ loop), which induce the conformational change of the β5^A^-β6^A^ loop. Subsequently, the hydrogen-bonding interaction between the main-chain amine of Glu125^A^ (the β5^A^–β6^A^ loop) and the main-chain carbonyl of Asn226^A^ (the α7^A^ helix) is disrupted and the N-terminal region of the α7^A^ helix undergoes conformational change to transform from the loop conformation to the helical conformation. Consequently, the side chain of Lys142^A^ (the β7^A^ strand) forms a tripartite hydrogen-bonding network with the side chains of Try228^A^ (the α7^A^ helix) and Asp181^A^ (the α5^A^ helix). The alterations of the hydrogen-bonding interactions among the β7^A^ strand, the β5^A^–β6^A^ loop and the α7^A^ helix further stabilize the conformational changes of the β5^A^–β6^A^ loop. As a result, the side chain of Tyr126^A^ (the β5–β6 loop) undergoes conformational change and assumes a conformation suitable for the ICT binding at the active site, and hence the partially activated state of the enzyme has a moderately decreased *S*_*0.5*,ICT_ and a moderately increased catalytic efficiency.

The α^Mg^γ^Mg+CIT+ADP^ structure represents the fully activated state of the enzyme. In this state, the ADP binding does not induce further conformational changes at the allosteric site and the active site, but establishes a more extensive hydrogen-bonding network among CIT, ADP and the surrounding residues through the metal ion and stabilizes the CIT and ADP binding with each other, which enhances the structural communication between the allosteric and active sites and further stabilizes the ICT binding at the active site. Therefore, the binding of CIT and ADP together has a synergistic activation effect, and the fully activated state of the enzyme has a substantially decreased *S*_*0.5*,ICT_ and a significant increased catalytic efficiency.

Yeast NAD-IDH consists of the IDH1/IDH2 heterodimer as the basic structural and functional unit, which is allosterically regulated by CIT and AMP^21^,^22^. The crystal structures of yeast NAD-IDH show that the binding of CIT at the allosteric site could induce conformational changes of the active site and thus enhances the binding affinity for ICT^21^. Yeast IDH1/IDH2 heterodimer has a similar structural topology as human αγ heterodimer. In addition, sequence alignment of yeast IDH1 and IDH2 subunits and human α and γ subunits shows that residues at the allosteric site, the active site, and the heterodimer interface are largely strictly conserved (Supplementary Figure S5). Thus, we performed a detailed structural comparison of yeast IDH1/IDH2 and human αγ heterodimers, which shows that the two heterodimers assume very similar structures at the allosteric site, the active site and the heterodimer interface in both the apo and CIT-bound structures; however, there are some conformational differences (Supplementary Figure S6). Specifically, in the apo IDH1/IDH2 heterodimer, residues 78–92 at the allosteric site assume a helical conformation which spatially occupies the CIT-binding site, and the N-terminal region of the α7 helix in both IDH1 and IDH2 subunits at the heterodimer interface adopts a helical conformation; the CIT binding induces structural change of residues 78–92 from the helical conformation to a loop conformation but does not induce structural change of the N-terminal region of the α7 helix. In the apo αγ heterodimer, residues 77–91 at the allosteric site assume a loop conformation which does not block the CIT-binding site, and the N-terminal region of the α7 helix in both α and γ subunits at the heterodimer interface assumes a loop conformation; the CIT binding does not induce structural change of residues 77–91 but induces structural change of the N-terminal region of the α7 helix from the loop conformation to a helical conformation.

Moreover, structural comparison of the apo and CIT-bound IDH1/IDH2 heterodimer shows that upon the CIT binding, the allosteric site, the active site and the heterodimer interface undergo conformational changes in similar manners as those in human αγ heterodimer (Supplementary Figure S7). In the apo IDH1/IDH2 structure, the CIT-binding site of the IDH1 subunit is spatially occupied by residues 78–92 with a helical conformation (Supplementary Figure S7a,b). Meanwhile, the side chain of Arg98^IDH1^ (corresponding to Arg97^G^) forms a cation-π interaction with the side chain of Phe136^IDH1^ (corresponding to Tyr135^G^), making the side chains of Arg98^IDH1^ and Phe136^IDH1^ point away from the CIT-binding site. At the heterodimer interface, the β5–β6 loop in both IDH1 and IDH2 subunits adopts a conformation similar to that in the α^Mg^γ structure and interacts with the N-terminal region of the α7 helix and the β7 strand via several hydrogen bonds; the β7 strand does not bend towards the α7 helix and there is no hydrogen-bonding interaction between the two structure elements (Supplementary Figures S6 and S7a,c,d). At the active site of the IDH2 subunit, the side chain of Tyr142^IDH2^ (corresponding to Tyr126^A^) assumes a similar conformation as that of Tyr126^A^ in the α^Mg^γ structure.

In the CIT-bound IDH1/IDH2 structure, residues 78–92 at the allosteric site adopt a loop conformation; the cation-π interaction between the side chains of Arg98^IDH1^ and Phe136^IDH1^ is disrupted, and consequently the side chain of Arg98^IDH1^ assumes a differed conformation to form a hydrogen bond with the γ-carboxyl of CIT, and the side chain of Phe136^IDH1^ is rotated towards and makes van der Waals contacts with the γ-carboxyl of CIT (Supplementary Figure S7a,b). In addition, the β5^IDH1^–β6^IDH1^ loop adopts a similar conformation as that in the α^Mg^γ^Mg+CIT^ structure and several residues of this loop change their hydrogen-bonding interactions with residues of the β7^IDH1^ strand (Supplementary Figures S6 and S7c,d). These changes lead to the formation of a tripartite hydrogen-bonding network among the side chains of Lys152^IDH1^ (corresponding to Lys151^G^), Tyr239^IDH1^ (corresponding to Tyr237^G^) and Asp191^IDH1^ (corresponding to Asp190^G^), which further facilitate and stabilize the conformational change of the β7^IDH1^ strand to bend towards the α7^IDH1^ helix. Similar to the αγ heterodimer, the conformational changes at the allosteric site and the heterodimer interface of the IDH1 subunit are transmitted to the IDH2 subunit and the active site in a pseudo symmetric manner. Through the extensive hydrogen-bonding interactions between the β7^IDH1^ strand and the β7^IDH2^ strand in the four-stranded β-sheet at the heterodimer interface, the β7^IDH2^ strand is induced to bend towards the α7^IDH2^ helix and concurrently the β5^IDH2^–β6^IDH2^ loop adopts a conformation similar to that in the α^Mg^γ^Mg+CIT^ structure (Supplementary Figure S7a). These conformational changes lead to the disruption of several hydrogen bonds and the establishment of a more extensive hydrogen-bonding interaction network among the β7^IDH2^ strand, the β5^IDH2^–β6^IDH2^ loop and the α7^IDH2^ helix (Supplementary Figure S7c,d). In particular, the side chain of Lys158^IDH2^ (corresponding to Lys142^A^) forms a tripartite hydrogen-bonding network with the side chains of Tyr246^IDH2^ (corresponding to Tyr228^A^) and Asp197^IDH2^ (corresponding to Asp181^A^). As a result, the side chain of Tyr142^IDH2^ (corresponding to Tyr126^A^) is rotated towards the active site and assumes a conformation favorable for ICT binding. Furthermore, similar to the αγ heterodimer, structural comparison between the CIT-bound and CIT+AMP-bound IDH1/IDH2 shows that the binding of CIT creates the AMP-binding site and the binding of AMP does not induce further conformational changes at the allosteric site and the active site.

These results together indicate that yeast IDH1/IDH2 heterodimer and human αγ heterodimer use a similar molecular mechanism for structural communication between the allosteric site and the active site, and thus share a common allosteric regulation mechanism. Furthermore, sequence alignment of human and yeast NAD-IDHs with other eukaryotic NAD-IDHs from *Caenorhabditis elegans, Arabidopsis thaliana, Danio rerio*, and *Xenopus laevis* shows that the residues composing the allosteric site, the active site and the heterodimer interface, and especially those involved in the conformational changes upon the binding of CIT (and ADP/AMP) are also largely strictly conserved (Supplementary Figure S5), suggesting that the other eukaryotic NAD-IDHs are likely to utilize a similar allosteric regulation mechanism as human αγ heterodimer.

## Methods

### Cloning, expression, and purification

The αγ heterodimer of human NAD-IDH was prepared as described previously^40^. Briefly, the DNA fragments encoding the α and γ subunits of human NAD-IDH were cloned into the co-expression vector pQlinkN with the C-terminal of the γ subunit attached with a TEV protease cleavage site and a His_6_ tag following the pQlink cloning procedure^41^. The pQlinkN-α-γ-tev-His_6_ plasmid was transformed into *E. coli* BL21(DE3) Codon-Plus strain (Novagen). When the culture of the transformed cells reached an OD_600_ of 0.5, the protein expression was induced by 0.4 mM IPTG for 20 hr at 23 °C. The bacterial cells were harvested, resuspended, and sonicated on ice in the lysis buffer [50 mM HEPES-Na, pH 7.4, 200 mM NaCl, 0.2 mM MnCl_2_, 10% (w/v) glycerol, and 7.2 mM β-ME] supplemented with 1 mM PMSF. The target protein was purified by affinity chromatography using a Ni-NTA column (Qiagen) with the lysis buffer supplemented with 20 mM and 200 mM imidazole serving as the washing and elution buffers, respectively. The elution fraction was dialyzed overnight against the lysis buffer supplemented with proper amount of TEV protease to lower the concentration of imidazole to <10 mM and to cleave the His_6_-tag of the target protein. The protein mixture was reloaded on a Ni-NTA column and washed with the lysis buffer supplemented with 10 mM imidazole. The flow-through fraction contains the target protein, which was further purified by gel filtration using a Superdex 200 10/300 GL column (GE Healthcare) equilibrated with the storage buffer (10 mM HEPES, pH 7.4, 200 mM NaCl, and 5 mM β-ME). The purity of the protein was assessed by 12% SDS-PAGE. The purified protein was concentrated to 10 mg/ml and stored for further structural and biochemical studies.

Mutants of the αγ heterodimer containing point mutations in the α and γ subunits were constructed using the QuikChange^®^ Site-Directed Mutagenesis kit (Strategene). Expression and purification of the mutants were carried out the same as for the wild-type protein.

### Enzymatic activity assay

The enzymatic activity of the αγ heterodimer was determined by monitoring the formation of NADH at 340 nm (*ε* = 6220 M^−1^cm^−1^) using a Coulter DU 800 spectrophotometer (Beckman) at 25 °C. The standard reaction solution (1 ml) consisted of 33 mM Tris-acetate, pH 7.4, 2 ng/ml enzyme, 80 mM _DL_-ICT, 2 mM Mn^2+^, and 3.2 mM NAD. The reaction was initiated by addition of NAD. The specific activity is defined as the amount of NADH produced per minute per milligram of enzyme (μmol/min/mg) at the standard conditions. Kinetic data in the absence of activators were measured at the standard conditions with varied concentrations of _DL_-ICT (0–80 mM) to obtain the *V*_max_ and *S*_*0.5*_ values for ICT. Kinetic data in the presence of activator(s) were determined at the above conditions in the presence of given concentrations of CIT or/and ADP. Kinetic parameters were obtained by fitting the data into the Non-Michaelis-Menten equation “*V* = *V*_max_*[S]^h/(*S*_*0.5*_^h + [S]^h)” using the Graphpad Prism program (Graphpad Software), where “h” is the Hill coefficient, “*S*_*0.5*_” is the apparent *K*m value for ICT (the ICT concentration at which the reaction rate is 0.5 * *V*_max_), and “[S]” is the concentration of ICT. All the experiments were performed in three independent measurements and the values were the averages of the three measurements with the standard errors.

### Crystallization and diffraction data collection

Crystallization was performed using the hanging drop vapor diffusion method at 20 °C by mixing equal volume (1 μl) of protein solution (10 mg/ml) and reservoir solution. Crystals of the αγ heterodimer without any activators bound to the γ subunit (α^Mg^γ) were grown from drops with the reservoir solution containing 0.1 M HEPES-Na, pH 7.5, 50 mM MgCl_2_, and 30% (v/v) PEGMME 550. Crystals of the αγ heterodimer with Mg^2+^ and CIT bound to the γ subunit (α^Mg^γ^Mg+CIT^) were grown from drops consisting of the protein solution supplemented with 0.2 mM Mg^2+^ and the reservoir solution containing 0.2 M sodium citrate, pH 8.0, and 20% (w/v) PEG3350. Crystals of the αγ heterodimer with Mg^2+^, CIT and ADP bound to the γ subunit (α^Mg^γ^Mg+CIT+ADP^) were grown from drops consisting of the protein solution supplemented with 0.2 mM Mg^2+^ and 2 mM ADP and the above reservoir solution. Crystals of the αγ heterodimer with Mg^2+^, ICT and ADP bound to the γ subunit (α^Mg^γ^Mg+ICT+ADP^) were grown from drops consisting of the protein solution supplemented with 0.2 mM Mg^2+^, 2 mM ICT and 2 mM ADP and the reservoir solution containing 0.1 M HEPES-Na, pH 7.5, and 12% (w/v) PEG 3350. Crystals of the mutant αγ_K151A_ heterodimer with Mg^2+^, CIT and ADP bound to the γ subunit (αγ_K151A_^Mg+CIT+ADP^) were grown at the same conditions as for the crystals of the α^Mg^γ^Mg+CIT+ADP^ heterodimer. Prior to diffraction data collection, the crystals were cryoprotected using the reservoir solution supplemented with 25% ethylene glycol and then flash-cooled into liquid N_2_. Diffraction data were collected at 100 K at BL19U1 of the National Facility for Protein Science in Shanghai and processed with HKL2000^42^. Statistics of the diffraction data are summarized in Table 1.

### Structure determination and refinement

All structures were determined with the molecular replacement (MR) method as implemented in program Phaser^43^. The α^Mg^γ structure was solved using the structure of HcIDH bound with NADP (PDB code 1T09)^14^ as the search model. The α^Mg^γ^Mg+CIT^ structure was solved using the α^Mg^γ structure as the search model, which was subsequently used as the search model to solve the α^Mg^γ^Mg+CIT+ADP^, α^Mg^γ^Mg+ICT+ADP^ and αγ_K151A_^Mg+CIT+ADP^ structures. Initial structure refinement was carried out with program Phenix^44^ and final structure refinement was performed with program REFMAC5^45^. Model building was performed with program Coot^46^. Stereochemistry and quality of the structure models were analyzed using programs in the CCP4 suite^47^ and the PISA server^48^. All structure figures were prepared using PyMol^49^. The structure-based sequence alignment figures were prepared using ESPpript 3.0^50^. Statistics of the structure refinement and the final structure models are summarized in Table 1.

## Additional Information

**Accession codes:** The α^Mg^γ, α^Mg^γ^Mg+CIT^, α^Mg^γ^Mg+CIT+ADP^, α^Mg^γ^Mg+ICT+ADP^, and αγ_K151A_^Mg+CIT+ADP^ structures have been deposited in the Protein Data Bank with accession codes 5GRH, 5GRI, 5GRE, 5GRL, and 5GRF, respectively.

**How to cite this article**: Ma, T. *et al*. Molecular mechanism of the allosteric regulation of the αγ heterodimer of human NAD-dependent isocitrate dehydrogenase. *Sci. Rep.* **7**, 40921; doi: 10.1038/srep40921 (2017).

**Publisher's note:** Springer Nature remains neutral with regard to jurisdictional claims in published maps and institutional affiliations.

## Supplementary Material

Supplementary Information

## Figures and Tables

**Table 1:** Statistics of X-ray diffraction data and structure refinement.

Structure	α^Mg^γ	α^Mg^γ^Mg+CIT^	α^Mg^γ^Mg+CIT+ADP^	α^Mg^γ^Mg+ICT+ADP^	αγ_K151A_^Mg+CIT+ADP^
Diffraction data					
Wavelength (Å)	1.0000	1.0000	1.0000	0.9792	0.9792
Space group	*P*3_1_21	*P*3_1_21	*P*3_1_21	*P*3_1_21	*P*3_1_21
*a* (Å)	118.4	112.0	104.9	111.2	114.3
*b* (Å)	118.4	112.0	104.9	111.2	114.3
*c* (Å)	143.2	145.0	146.3	145.5	143.9
Resolution (Å)	19.71–2.80 (2.90–2.80)^a^	50.0–2.30 (2.38–2.30)	50.0–2.65 (2.74–2.65)	50.0–2.80 (2.90–2.80)	50.0–2.50 (2.59–2.50)
Observed reflections	309,587	906,555	250,203	72,179	187,289
Unique reflections (I/σ(I)>0)	28,718	46,471	27,500	25,878	38,210
Average redundancy	10.8 (10.6)	19.5 (14.4)	9.1 (9.2)	2.8 (2.8)	4.9 (5.0)
Average I/σ(I)	9.6 (4.4)	53.7 (3.9)	22.4 (4.4)	14.5 (2.5)	23.4 (2.9)
Completeness (%)	98.9 (100.0)	98.4 (86.6)	99.9 (100.0)	98.1 (99.6)	99.9 (99.9)
Rmerge (%)^b^	15.6 (48.2)	6.2 (45.8)	10.7 (53.7)	8.0 (49.8)	7.5 (45.7)
Refinement and structure model					
No. of reflections (*Fo* > 0σ(*Fo*))					
Working set	25,865	38,317	24,769	22,943	34,470
Test set	1,361	2,017	1,304	1,208	1,814
R factor/Free R factor (%)^c^	22.4/26.0	24.0/27.1	19.4/24.1	22.6/27.4	20.6/24.8
Total protein atoms	5,064	5,039	5,042	5,035	5,046
Total ligand atoms	1	15	42	42	41
Total solvent atoms	0	111	58	0	127
Average B factor (Å^2^)	59.0	44.3	49.3	39.7	68.6
Protein	59.1	44.9	49.6	39.9	68.9
ICT or CIT	—	20.5	30.6	33.9	52.0
ADP	—	—	38.5	17.1	84.7
Mg (active site)	27.8	32.9	49.5	23.6	—
Mg (allosteric site)	—	28.2	23.5	10.0	69.1
Water	—	19.7	32.3	—	55.5
RMS deviations					
Bond lengths (Å)	0.006	0.005	0.009	0.006	0.007
Bond angles (°)	1.1	1.0	1.3	1.1	1.3
Ramachandran plot (%)					
Most favored	90.2	90.4	89.5	89.3	90.0
Allowed	9.6	9.1	10.0	10.5	9.8
Generously allowed	0.2	0.5	0.5	0.2	0.2
Disallowed	0.0	0.0	0.0	0.0	0.0

^a^Numbers in parentheses refer to the highest resolution shell.

^b^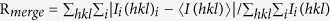.

^c^R factor = ∑||F_o_| − |F_c_||/∑|F_o_|.

**Table 2:** Effects of mutations of key residues on the specific activity and kinetic parameters of the αγ heterodimer.

Enzyme	Specific activity a(*μmol*/min/*mg*)	*S*_*0.5,ICT*_(*mM*)	Hill coefficient for ICT	*kcat/S*_*0.5,ICT*_ (*s*^*−1*^*mM*^*−1*^)
		−Activators/+activators	−Activators/+activators	−Activators/+activators
Residues at the CIT-binding subsite				
		−CIT/+5 mM CIT	−CIT/+5 mM CIT	−CIT/+5 mM CIT
Wild-type	7.27 ± 0.21	4.49 ± 0.15/0.589 ± 0.026	2.0 ± 0.1/1.0 ± 0.1	2.16 ± 0.03/27.8 ± 0.6
α-K173A	1.87 ± 0.19	6.38 ± 1.27/4.51 ± 0.26	1.2 ± 0.1/1.1 ± 0.1	0.38 ± 0.03/0.54 ± 0.05
α-N175A	1.27 ± 0.06	5.80 ± 0.84/2.60 ± 0.28	1.2 ± 0.1/1.0 ± 0.1	0.29 ± 0.01/0.65 ± 0.03
γ-N78A	8.07 ± 0.06	5.05 ± 0.85/0.532 ± 0.024	1.7 ± 0.1/1.0 ± 0.1	2.12 ± 0.02/32.1 ± 0.4
γ-T81A	4.13 ± 0.14	7.13 ± 0.34/6.16 ± 0.51	1.1 ± 0.1/1.1 ± 0.1	0.77 ± 0.03/1.01 ± 0.02
γ-S91A	6.70 ± 0.19	6.08 ± 0.27/1.51 ± 0.26	1.6 ± 0.1/1.3 ± 0.1	1.56 ± 0.04/13.9 ± 0. 6
γ-N93A	3.60 ± 0.08	6.08 ± 0.57/3.23 ± 0.22	1.2 ± 0.1/1.0 ± 0.1	0.78 ± 0.02/1.47 ± 0. 03
γ-R97A	1.13 ± 0.04	4.51 ± 0.43/4.28 ± 0.26	1.0 ± 0.1/1.1 ± 0.1	0.33 ± 0.01/0.34 ± 0.01
γ-R128A	1.67 ± 0.09	8.57 ± 0.42/7.34 ± 0.26	1.1 ± 0.1/1.0 ± 0.1	0.26 ± 0.01/0.30 ± 0.02
γ-Y135F	1.87 ± 0.05	8.80 ± 0.54/6.27 ± 0.28	1.1 ± 0.1/1.0 ± 0.1	0.27 ± 0.01/0.38 ± 0.01
γ-R272A	3.21 ± 0.06	19.3 ± 1.0/21.8 ± 1.6	1.0 ± 0.1/1.0 ± 0.1	0.21 ± 0.01/0.17 ± 0.00
Residues at the ADP-binding subsite				
		**−ADP/+1 mM ADP**	**−ADP/+1 mM ADP**	**−ADP/+1 mM ADP**
Wild-type	7.27 ± 0.21	4.49 ± 0.15/1.69 ± 0.05	2.0 ± 0.1/1.6 ± 0.1	2.16 ± 0.03/7.43 ± 0.06
γ-N273A	7.14 ± 0.36	6.39 ± 0.24/5.52 ± 0.22	1.5 ± 0.1/1.5 ± 0.1	1.43 ± 0.08/1.71 ± 0.09
γ-T274A	6.66 ± 0.23	6.85 ± 0.15/6.32 ± 0.27	1.6 ± 0.1/1.5 ± 0.1	1.29 ± 0.04/1.35 ± 0.05
γ-K276A	6.02 ± 0.12	7.42 ± 0.15/2.04 ± 0.12	1.7 ± 0.1/1.5 ± 0.1	1.07 ± 0.03/5.73 ± 0.15
γ-N285A	0.02 ± 0.01	ND	ND	ND
Residues at the heterodimer interface				
		**−CIT-ADP/+CIT+ADP**^b^	**−CIT-ADP/+CIT+ADP**	**−CIT-ADP/+CIT+ADP**
Wild-type	7.27 ± 0.21	4.49 ± 0.15/0.18 ± 0.02	2.0 ± 0.1/1.0 ± 0.1	2.16 ± 0.03/96.7 ± 2.7
α-E125A	3.80 ± 0.12	5.00 ± 0.47/0.15 ± 0.01	1.7 ± 0.1/1.0 ± 0.1	1.01 ± 0.04/52.8 ± 2.1
α-K142A	1.66 ± 0.01	5.19 ± 0.35/4.09 ± 0.33	1.0 ± 0.1/1.1 ± 0.1	0.43 ± 0.02/0.37 ± 0.01
α-D181A	0.04 ± 0.01	ND	ND	ND
α-Y228F	0.50 ± 0.01	14.5 ± 0.9/6.02 ± 0.16	1.1 ± 0.1/1.0 ± 0.1	0.046 ± 0.002/0.208 ± 0.012
γ-E134A	7.51 ± 0.31	4.63 ± 0.32/0.16 ± 0.01	1.7 ± 0.1/1.1 ± 0.1	2.19 ± 0.02/118 ± 2
γ-K151A	0.53 ± 0.01	8.12 ± 0.16/9.01 ± 0.71	1.1 ± 0.1/1.0 ± 0.1	0.090 ± 0.002/0.065 ± 0.003
γ-D190A	5.43 ± 0.12	10.2 ± 0.6/12.0 ± 0.7	1.1 ± 0.1/1.0 ± 0.1	0.73 ± 0.04/0.61 ± 0.02
γ-Y237F	2.81 ± 0.02	14.8 ± 0.5/17.3 ± 0.9	1.0 ± 0.1/1.0 ± 0.1	0.25 ± 0.01/0.41 ± 0.02

^a^The specific activity was determined at the standard conditions as described in Methods. The *S*_*0.5*,ICT_ and Hill coefficient for ICT were determined at the standard conditions with varied concentrations of ICT. A molecular mass of 80 kDa was used to calculate the mole of the αγ heterodimer per mg of protein (1.25 × 10^−8^ mol of the heterodimer/mg of protein).

^b^The concentrations of CIT and ADP were both 1 mM.
