# Supplementary material for: Molecular mechanism of the allosteric regulation of the αγ heterodimer of human NAD-dependent isocitrate dehydrogenase
Source: Sci Rep. 2017 Jan 18;7:40921. doi: 10.1038/srep40921 (PMC5241874; doi:10.1038/srep40921)
Supplement: Supplementary Information [file srep40921-s1.pdf]

SUPPLEMENTARY INFORMATION

Molecular mechanism of the allosteric regulation of the αγ heterodimer of human NAD-dependent isocitrate dehydrogenase

Tengfei Ma[1], Yingjie Peng[1], Wei Huang[1], and Jianping Ding[1,2,3,4,*]

[1] National Center for Protein Science Shanghai, State Key Laboratory of Molecular Biology, Center for Excellence in Molecular Cell Science, Institute of Biochemistry and Cell Biology, Shanghai Institutes for Biological Sciences, Chinese Academy of Sciences, 320 Yueyang Road, Shanghai 200031, China

[2] School of Life Science and Technology, ShanghaiTech University, 100 Haike Road, Shanghai 201210, China

[3] Shanghai Science Research Center, Chinese Academy of Sciences, 333 Haike Road, Shanghai 201210, China

[4] Collaborative Innovation Center for Genetics and Development, Fudan University, 2005 Songhu Road, Shanghai 200438, China

*To whom correspondence should be addressed: Jianping Ding (Phone: 86-21-5492-1619, E-mail: jpding@sibcb.ac.cn).

Table S1. Pair-wise superposition of the $\alpha\gamma$ heterodimer in different structures.

Overall RMSD (Å)*	$\alpha^{Mg}\gamma$	$\alpha^{Mg}\gamma^{Mg+CIT}$	$\alpha^{Mg}\gamma^{Mg+CIT+ADP}$	$\alpha^{Mg}\gamma^{Mg+ICT+ADP}$
$\alpha^{Mg}\gamma^{Mg+CIT}$	1.2 (652)	----		
$\alpha^{Mg}\gamma^{Mg+CIT+ADP}$	1.0 (654)	0.6 (659)	----	
$\alpha^{Mg}\gamma^{Mg+ICT+ADP}$	1.2 (653)	0.4 (659)	0.6 (659)	----
$\alpha\gamma_{K151A}{}^{Mg+CIT+ADP}$	0.8 (657)	0.8 (654)	0.8 (658)	0.8 (653)

* Numbers in parentheses are the numbers of Cα atoms used in the superposition.

Figure S1. Representative simulated annealing composite omit 2*Fo-Fc* maps at the active and allosteric sites of the αγ heterodimer in different structures.

(a) The active site in the $\alpha^{Mg}\gamma$ structure (contoured at 1.0σ level). (b) The active site in the $\alpha^{Mg}\gamma^{Mg+CIT}$ structure (contoured at 2.0σ level). (c) The allosteric site in the $\alpha^{Mg}\gamma^{Mg+CIT}$ structure (contoured at 2.0σ level). (d) The allosteric site in the $\alpha^{Mg}\gamma^{Mg+CIT+ADP}$ structure (contoured at 1.0σ level). (e) The allosteric site in the $\alpha^{Mg}\gamma^{Mg+ICT+ADP}$ structure (contoured at 1.0σ level). (f) The allosteric site in the $\alpha\gamma_{K151A}{}^{Mg+CIT+ADP}$ structure (contoured at 1.0σ level). The residues and ligands are shown with ball-and-stick models, and the Mg^{2+} and water molecules are shown with green and red spheres, respectively. The coordination bonds of the metal ion are indicated with dashed lines.

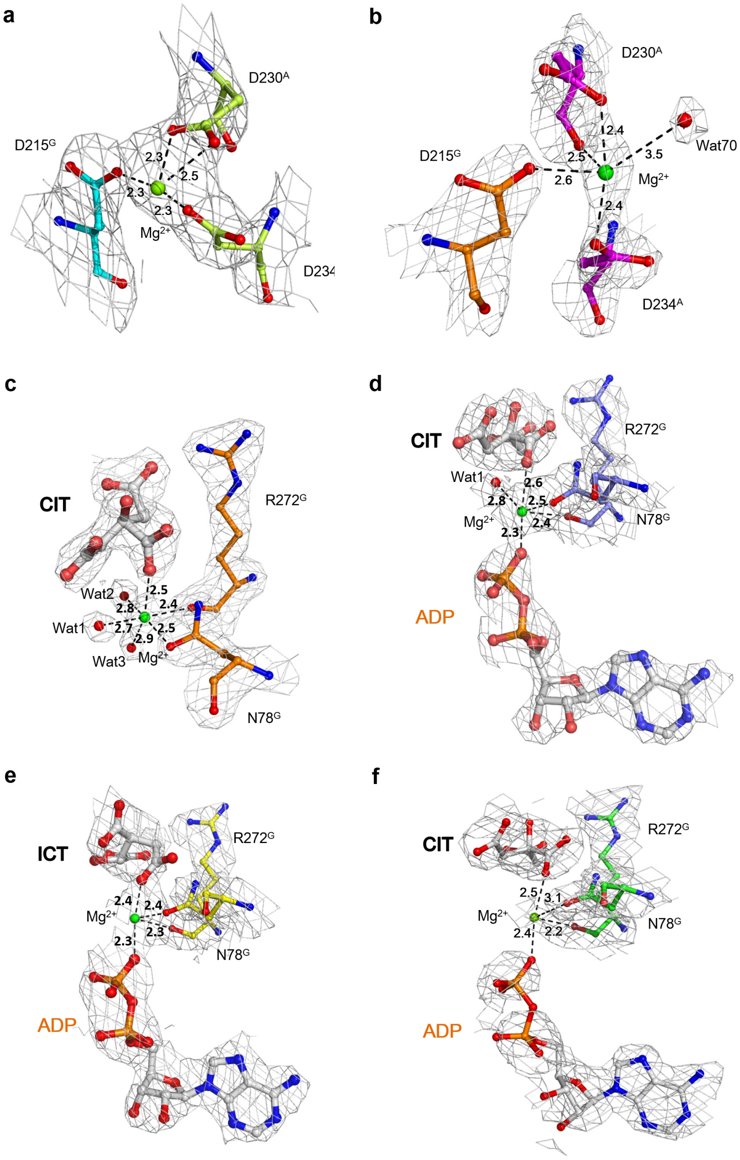

Figure S2. Structure-based sequence alignment of the α and γ subunits of human NAD-IDH with several representative IDHs. The sequences of other IDHs included in the alignment are: the IDH1 and IDH2 subunits of *S. cerevesiae* NAD-IDH (ScIDH1 and ScIDH2, PDB code 3BLX), human cytosolic NADP-IDH (HcIDH, PDB code 1T0L), porcine mitochondrial NADP-IDH (PmIDH, PDB code 1LWD), and *E. coli* NADP-IDH (EcIDH, PDB code 1IKB). Invariant residues are highlighted by shaded red boxes and conserved residues by open blue boxes. The secondary structures of these enzymes are placed on the top of the alignment.

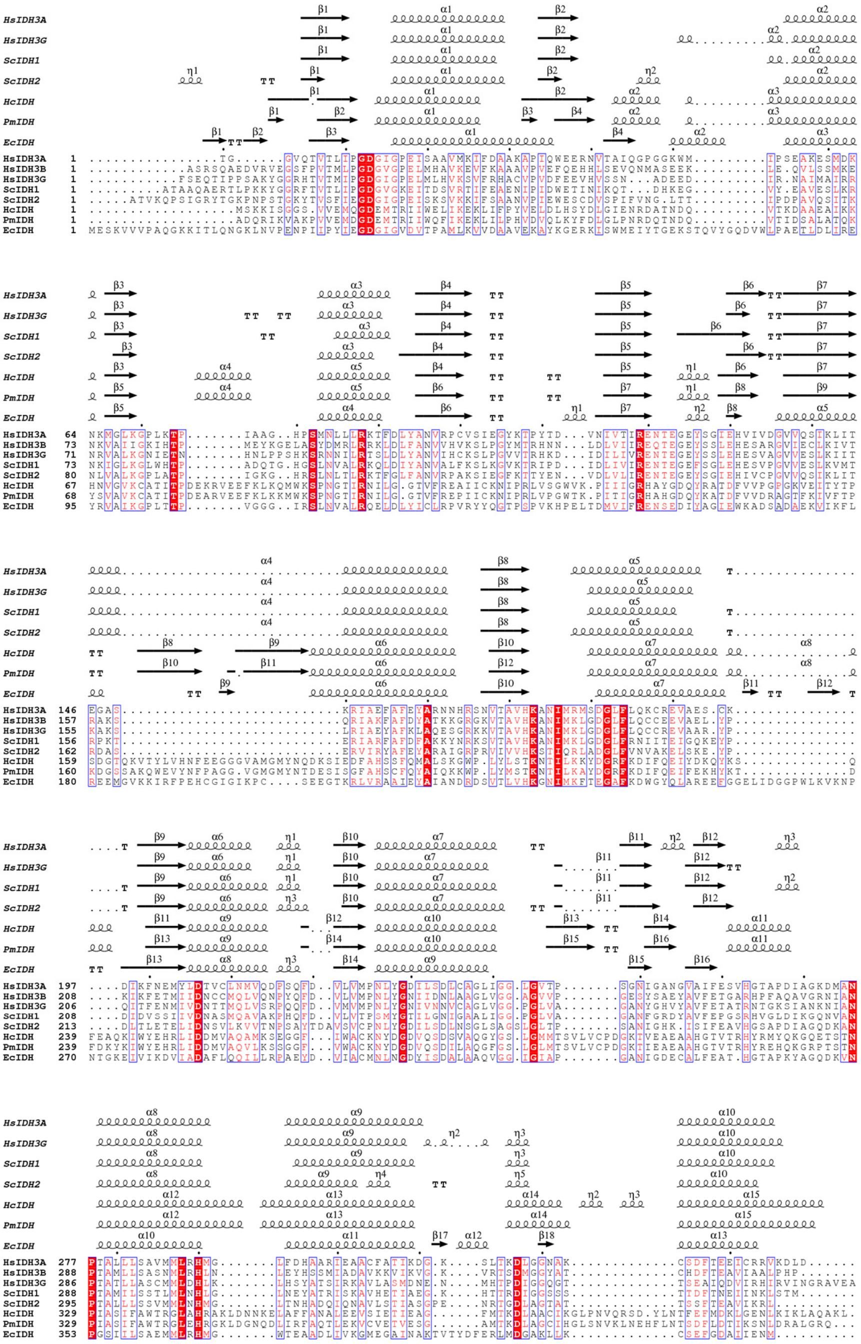

Figure S3. Comparison of the active sites of the $\alpha^{Mg}\gamma^{Mg+CIT}$ structure (orange), the PmIDH^{Mn+ICT} structure (porcine mitochondrial NADP-IDH, PDB code 1LWD, magenta), and the HcIDH$^{Ca+ICT+NADP}$ structure (human cytosolic NADP-IDH, PDB code 1T0L, blue). For clarity, only the hydrogen-bonding interactions of ICT with the surrounding residues in the PmIDH^{Mn+ICT} structure are shown.

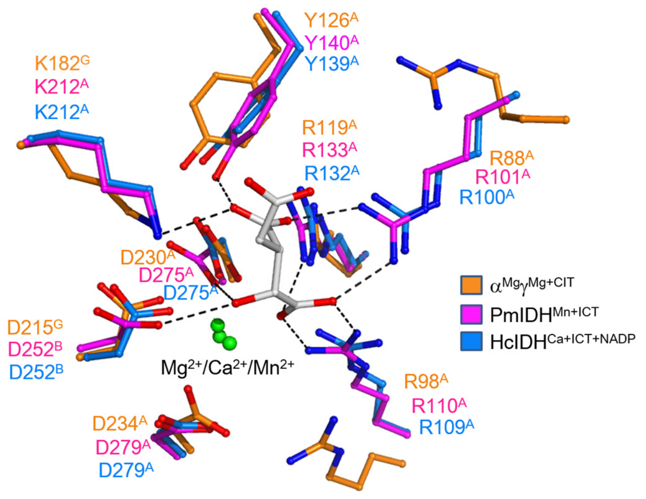

Figure S4. Comparison of the αγ heterodimer in different structures.
(a) Comparison of the allosteric site, the active site, and the structure elements at the heterodimer interface in the $\alpha^{Mg}\gamma^{Mg+CIT}$ (orange), $\alpha^{Mg}\gamma^{Mg+CIT+ADP}$ (slate), $\alpha^{Mg}\gamma^{Mg+ICT+ADP}$ (yellow), and $\alpha\gamma_{K151A}{}^{Mg+CIT+ADP}$ (green) structures. The orientations of the α6 and α7 helices in the α and γ subunits are indicated with dashed arrows. Some key residues involved in the conformational changes are shown with side chains. (b) Comparison of the CIT-binding subsite in the $\alpha^{Mg}\gamma^{Mg+CIT}$ (orange), $\alpha^{Mg}\gamma^{Mg+CIT+ADP}$ (slate), $\alpha^{Mg}\gamma^{Mg+ICT+ADP}$ (yellow), and $\alpha\gamma_{K151A}{}^{Mg+CIT+ADP}$ (green) structures. The bound CIT (or ICT) is shown with a ball-and-stick model, the Mg^{2+} with a green sphere, and the surrounding residues with side chains. (c) Comparison of the active site in the $\alpha^{Mg}\gamma$ (cyan), $\alpha^{Mg}\gamma^{Mg+CIT}$ (orange), $\alpha^{Mg}\gamma^{Mg+CIT+ADP}$ (slate), $\alpha^{Mg}\gamma^{Mg+ICT+ADP}$ (yellow), and $\alpha\gamma_{K151A}{}^{Mg+CIT+ADP}$ (green) structures. The Mg^{2+} is shown with a green sphere and the surrounding residues with side chains.

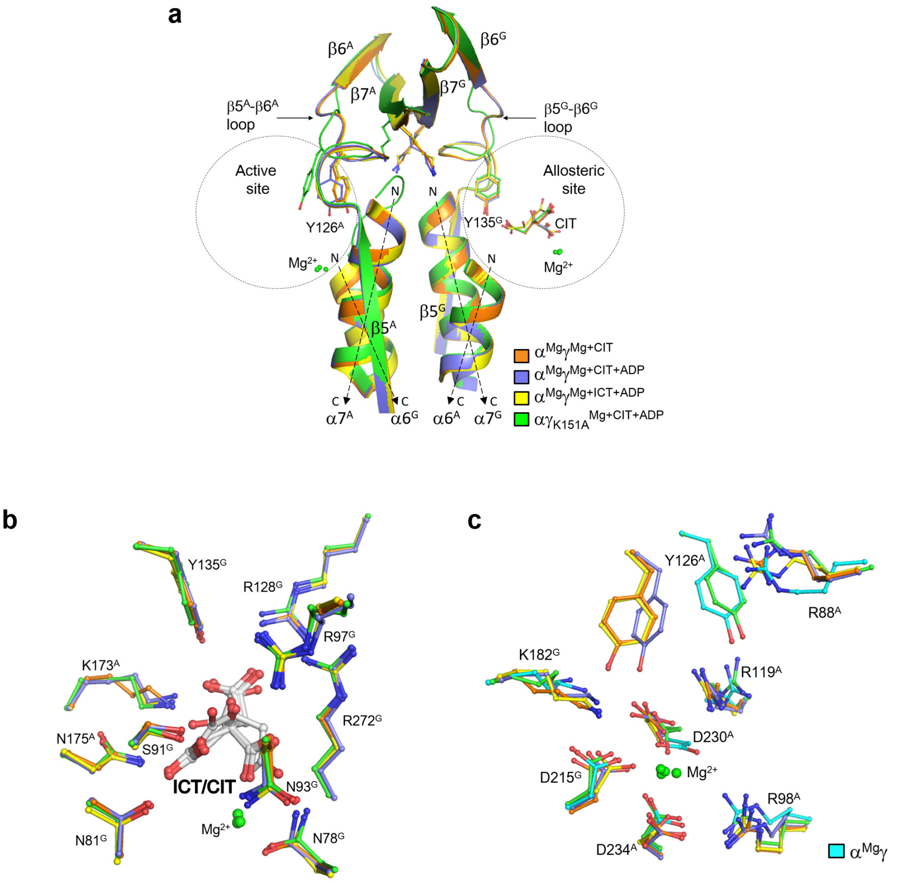

Figure S5. Sequence alignment of NAD-IDHs from different eukaryotic species. The sequences included in the alignment are: the α, β, γ subunits of human NAD-IDH (HsIDH3A, HsIDH3B, and HsIDH3G); the IDH1 and IDH2 subunits of *Saccharomyces cerevisiae* NAD-IDH (ScIDH1 and ScIDH2); the α, β, γ1 and γ2 subunits of *Caenorhabditis elegans* NAD-IDH (CeIDH3A, HsIDH3B, HsIDH3G1, and HsIDH3G2); the IDH1, IDH2, IDH3, IDH5 and IDH6 subunits of *Arabidopsis thaliana* NAD-IDH (AtIDH1, AtIDH2, AtIDH3, AtIDH5, and AtIDH6); the α, β and γ subunits of *Danio rerio* NAD-IDH (DrIDH3A, DrIDH3B, and DrIDH3G); and the α, β and γ subunits of *Xenopus laevis* NAD-IDH (XlIDH3A, XlIDH3B, and XlIDH3G). Invariant residues are highlighted by shaded red boxes and conserved residues by open blue boxes. The residues corresponding to those composing the allosteric site and the active site in the αγ heterodimer of human NAD-IDH are indicated with red triangles, and those involved in the conformational changes at the heterodimer interface with blue stars.

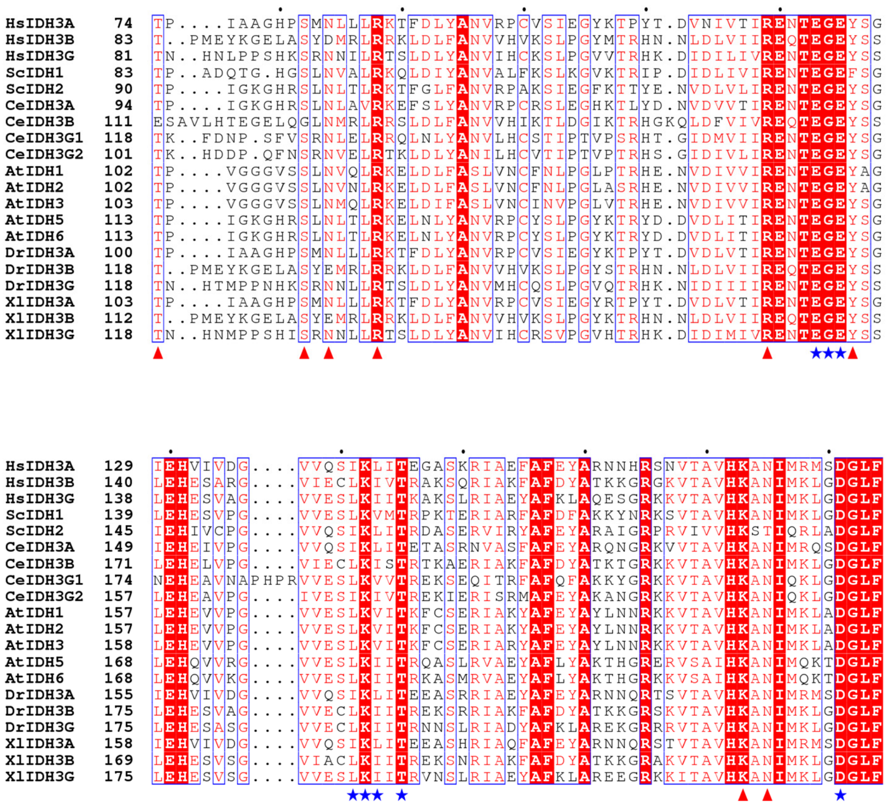

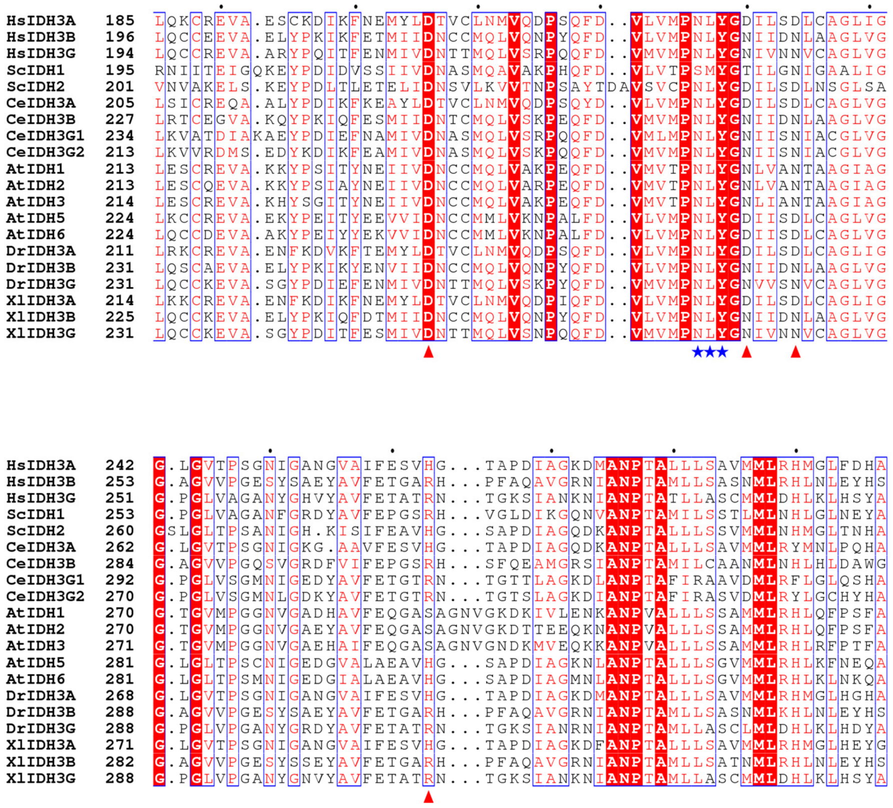
HsIDH3A 185 LQKCREVA.ESCKDIKFNEMYLDTVCLNMVQDPSQFD..VLVMPNLYGDILSDLCAGLIG
HsIDH3B 196 LQCCEEVA.ELYPKIKFETMIIDNCCMQLVQNPYQFD..VLVMPNLYGNIIDNLAAGLVG
HsIDH3G 194 LQCCREVA.ARYPQITFENMIVDNTTMQLVSRPQQFD..VMVMPNLYGNIVNNVCAGLVG
ScIDH1 195 RNIITEIGQKEYPDIDVSSIIVDNASMQAVAKPHQFD..VLVTPSMYGTILGNIGAALIG
ScIDH2 201 VNVAKELS.KEYPDLTLETELIDNSVLKVVTNPSAYTDAVSVCPNLYGDILSDLNSGLSA
CeIDH3A 205 LSICREQA.ALYPDIKFKEAYLDTVCLNMVQDPSQYD..VLVMPNLYGDILSDLCAGLVG
CeIDH3B 227 LRTCEGVA.KQYPKIQFESMIIDNTCMQLVSKPEQFD..VMVMPNLYGNIIDNLAAGLVG
CeIDH3G1 234 LKVATDIAKAEYPDIEFNAMIVDNASMQLVSRPQQFD..VMLMPNLYGNIISNIACGLVG
CeIDH3G2 213 LKVVRDMS.EDYKDIKFEAMIVDNASMQLVSKPQQFD..VMVMPNLYGNIISNIACGLVG
AtIDH1 213 LESCREVA.KKYPSITYNEIIVDNCCMQLVAKPEQFD..VMVTPNLYGNLVANTAAGIAG
AtIDH2 213 LESCQEVA.KKYPSIAYNEIIVDNCCMQLVARPEQFD..VMVTPNLYGNLVANTAAGIAG
AtIDH3 214 LESCREVA.KHYSGITYNEIIVDNCCMQLVAKPEQFD..VMVTPNLYGNLIANTAAGIAG
AtIDH5 224 LKCCREVA.EKYPEITYEEVVIDNCCMMLVKNPALFD..VLVMPNLYGDIISDLCAGLVG
AtIDH6 224 LQCCDEVA.AKYPEIYYEKVVIDNCCMMLVKNPALFD..VLVMPNLYGDIISDLCAGLVG
DrIDH3A 211 LRKCREVA.ENFKDVKFTEMYLDTVCLNMVQDPSQFD..VLVMPNLYGDILSDLCAGLIG
DrIDH3B 231 LQSCAEVA.ELYPKIKYENVIIDNCCMQLVQNPYQFD..VLVMPNLYGNIIDNLAAGLVG
DrIDH3G 231 LQCCKEVA.SGYPDIEFENMIVDNTTMQLVSKPYQFD..VMVMPNLYGNVVSNVCAGLVG
XlIDH3A 214 LKKCREVA.ENFKDIKFNEMYLDTVCLNMVQDPIQFD..VLVMPNLYGDILSDLCAGLIG
XlIDH3B 225 LQCCKEVA.ELYPKIQFDTMIIDNCCMQLVQNPYQFD..VLVMPNLYGNIIDNLAAGLVG
XlIDH3G 231 LQCCKEVA.SGYPDITFESMIVDNTTMQLVSNPQQFD..VMVMPNLYGNIVNNVCAGLVG
★★★
HsIDH3A 242 G.LGVTPSGNIGANGVAIFESVHG...TAPDIAGKDMANPTALLLSAVMMLRHMGLFDHA
HsIDH3B 253 G.AGVVPGESYSAEYAVFETGARH...PFAQAVGRNIANPTAMLLSASNMLRHLNLEYHS
HsIDH3G 251 G.PGLVAGANYGHVYAVFETATRN...TGKSIANKNIANPTATLLASCMMLDHLKLHSYA
ScIDH1 253 G.PGLVAGANFGRDYAVFEPGSRH...VGLDIKGQNVANPTAMILSSTLMLNHLGLNEYA
ScIDH2 260 GSLGLTPSANIGH.KISIFEAVHG...SAPDIAGQDKANPTALLLSSVMMLNHMGLTNHA
CeIDH3A 262 G.LGVTPSGNIGKG.AAVFESVHG...TAPDIAGQDKANPTALLLSAVMMLRYMNLPQHA
CeIDH3B 284 G.AGVVPGQSVGRDFVIFEPGSRH...SFQEAMGRSIANPTAMILCAANMLNHLHLDAWG
CeIDH3G1 292 G.PGLVSGMNIGEDYAVFETGTRN...TGTTLAGKDLANPTAFIRAAVDMLRFLGLQSHA
CeIDH3G2 270 G.PGLVSGMNLGDKYAVFETGTRN...TGTSLAGKDIANPTAFIRASVDMLRYLGCHYHA
AtIDH1 270 G.TGVMPGGNVGADHAVFEQGASAGNVGKDKIVLENKANPVALLLSSAMMLRHLQFPSFA
AtIDH2 270 G.TGVMPGGNVGAEYAVFEQGASAGNVGKDTTEEQKNANPVALLLSSAMMLRHLQFPSFA
AtIDH3 271 G.TGVMPGGNVGAEHAIFEQGASAGNVGNDKMVEQKKANPVALLLSSAMMLRHLRFPTFA
AtIDH5 281 G.LGLTPSCNIGEDGVALAEAVHG...SAPDIAGKNLANPTALLLSGVMMLRHLKFNEQA
AtIDH6 281 G.LGLTPSMNIGEDGIALAEAVHG...SAPDIAGMNLANPTALLLSGVMMLRHLKLNKQA
DrIDH3A 268 G.LGVTPSGNIGANGVAIFESVHG...TAPDIAGKDMANPTALLLSAVMMLRHMGLHGHA
DrIDH3B 288 G.AGVVPGESYSAEYAVFETGARH...PFAQAVGRNIANPTAMLLSASNMLKHLNLEYHS
DrIDH3G 288 G.PGLVPGANYGRDYAVFETATRN...TGKSIANRNIANPTAMLLASCLMLDHLKLHDYA
XlIDH3A 271 G.LGVTPSGNIGANGVAIFESVHG...TAPDIAGKDFANPTALLLSAVMMLRHMGLHEYG
XlIDH3B 282 G.AGVVPGESYSSEYAVFETGARH...PFAQAVGRNIANPTAMLLSATNMLRHLNLEYHS
XlIDH3G 288 G.PGLVPGANYGNVYAVFETATRN...TGKSIANKNIANPTAMLLASCMMLDHLKLHSYA

Figure S6. Structural comparison of the αγ heterodimer of human NAD-IDH and the IDH1/IDH2 heterodimer of yeast NAD-IDH. Structural comparison of human αγ heterodimer and yeast IDH1/IDH2 heterodimer at the allosteric site, the active site, and the heterodimer interface in the apo form (top panel) and the CIT-bound form (bottom panel). The α and γ subunits in the $\alpha^{Mg}\gamma$ structure are colored in lemon and cyan, and these in the $\alpha^{Mg}\gamma^{Mg+CIT}$ structure in magenta and orange, respectively. The IDH1 and IDH2 subunits in the apo structure **(**PDB code: 3BLX) are colored in pink and slate, and these in the CIT-bound structure **(**PDB code: 3BLV) in green and yellow, respectively. For easy comparison, we use the same secondary structure nomenclature for both human αγ heterodimer and yeast IDH1/IDH2 heterodimer. The structure elements and residues of the IDH1 and IDH2 subunits are superscripted by "1" and "2", respectively. The key residues are shown with side chains. The orientations of the α6 and α7 helices in both subunits are indicated with dashed arrows.

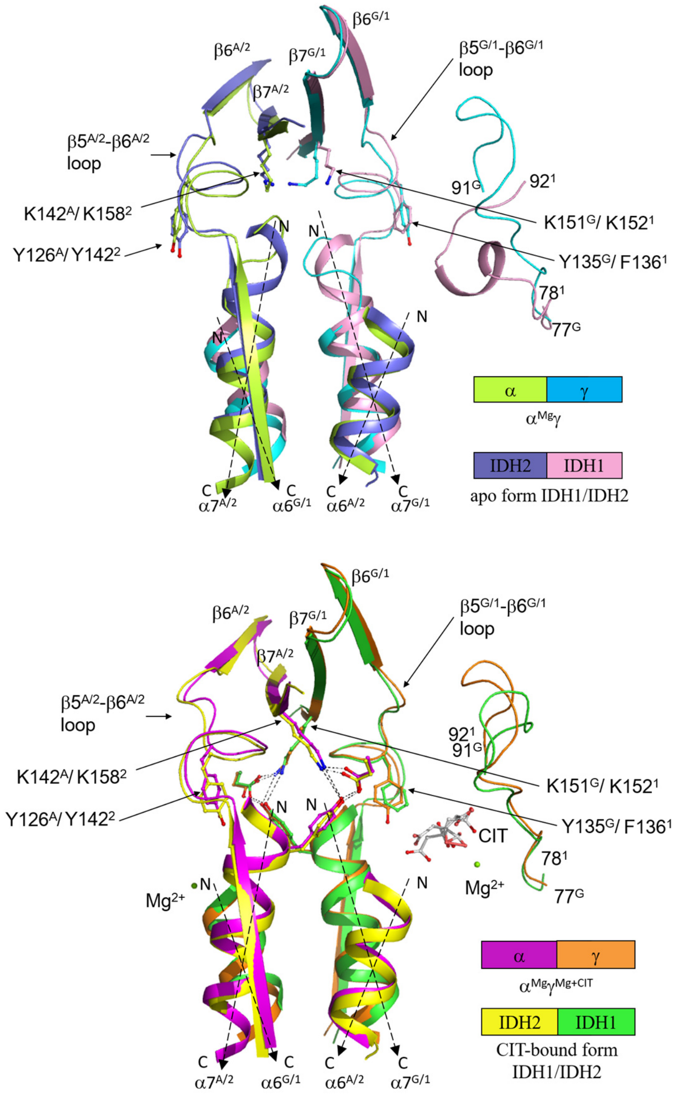

Figure S7. Structural comparison of the apo and CIT-bound IDH1/IDH2 heterodimer of yeast NAD-IDH.

(a) Comparison of the apo and CIT-bound IDH1/IDH2 structures at the allosteric site, the active site, and the heterodimer interface. The IDH1 and IDH2 subunits in the apo structure are colored in pink and slate, respectively; and these in the CIT-bound structure are colored in green and yellow, respectively. For easy comparison, we use the same secondary structure nomenclature for both human αγ heterodimer and yeast IDH1/IDH2 heterodimer. The structure elements and residues of the IDH1 and IDH2 subunits are superscripted by “1” and “2”, respectively. The key residues are shown with side chains. Upon the binding of CIT, significant conformational changes are observed at the residues 78^1-92^1 of IDH1, the C-terminal region of the β5-β6 loop and the β7 strand of both the IDH1 and IDH2 subunits at the heterodimer interface. The orientations of the α6 and α7 helices in both subunits are indicated with dashed arrows. The zoom-in panel on the right top shows the conformational changes of the $\beta7^1$ and $\beta7^2$ strands. For clarity, only the hydrogen-bonding interactions between the main chains of the residues are shown and the side chains are omitted. The $\beta7^A$ and $\beta7^G$ strands bend towards the $\alpha6^1$-$\alpha7^1$ and $\alpha6^1$-$\alpha7^1$ four-helix bundle, with the Cα atoms of $Ser150^1$, $Leu151^1$ and $Lys152^1$ of the $\beta7^1$ strand and $Ser156^2$, $Ile157^2$ and $Lys158^2$ of the $\beta7^2$ strand (indicated with black arrows) shifting about 2Å.

(b) Structure of the allosteric site in the apo (left panel) and CIT-bound (right panel) IDH1/IDH2 structures. In the apo structure, residues 78-92 adopt a helical conformation which block the CIT-binding site; in the CIT-bound structure, residues 78-92 adopt a loop conformation. The hydrophilic interactions between CIT and the surrounding residues are indicated with dashed lines and several key residues are shown with side chains.

(c) Structure of the heterodimer interface in the apo (left panel) and CIT-bound (right panel) IDH1/IDH2 structures. The structure elements at the heterodimer interface that undergo major conformational changes upon the CIT binding include the β5-β6 loop and the β7 strand of both IDH1 and IDH2 subunits. Several key residues are shown with side chains. For clarity, only the hydrogen-bonding interactions that are altered upon the CIT binding are indicated with dashed lines.

(d) A schematic diagram showing the hydrogen-bonding interactions among the β5-β6 loop, the α7 helix, the β7 strand, and the α5 helix of both IDH1 and IDH2 subunits in the apo and CIT-bound IDH1/IDH2 structures. The interactions in the apo structure are indicated with green lines; these disrupted upon the CIT binding are indicated with dashed green lines, and the newly formed interactions are indicated with red lines.

a

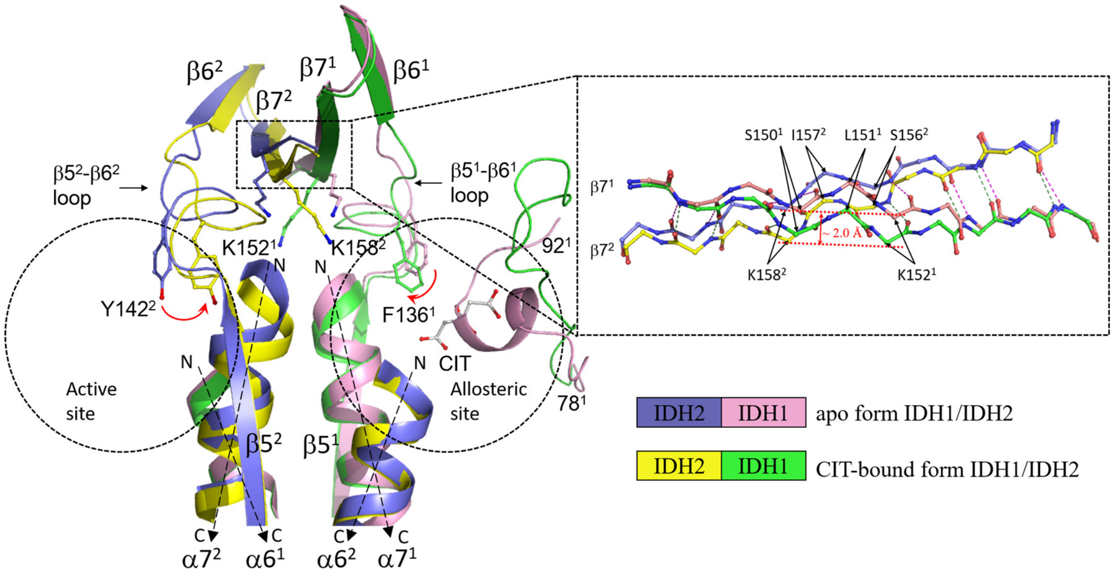
$\beta6^2$
$\beta7^1$
$\beta6^1$
$\beta7^2$
$\beta5^2$-$\beta6^2$ loop
$\beta5^1$-$\beta6^1$ loop
$K152^1$
$K158^2$
92^1
$Y142^2$
$F136^1$
CIT
Active site
Allosteric site
78^1
N
C
$\beta5^2$
$\beta5^1$
$\alpha7^2$
$\alpha6^1$
$\alpha6^2$
$\alpha7^1$
$S150^1$ $I157^2$ $L151^1$ $S156^2$
$K158^2$
$K152^1$
~ 2.0 Å
IDH2 IDH1 apo form IDH1/IDH2
IDH2 IDH1 CIT-bound form IDH1/IDH2

b

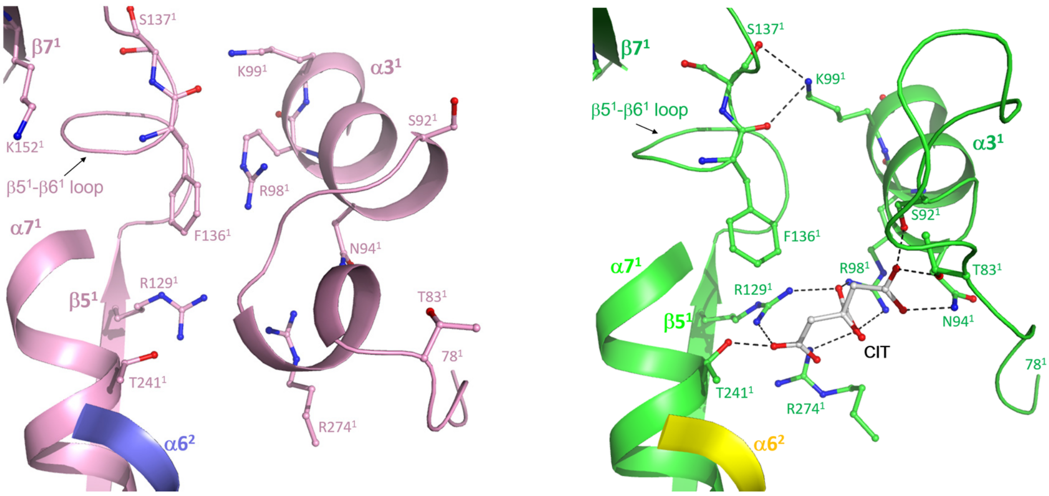
$\beta7^1$
$S137^1$
$K99^1$
$\alpha3^1$
$S92^1$
$K152^1$
$\beta5^1$-$\beta6^1$ loop
$R98^1$
$\alpha7^1$
$F136^1$
$N94^1$
$R129^1$
$\beta5^1$
$T83^1$
78^1
$T241^1$
$R274^1$
$\alpha6^2$
CIT

c

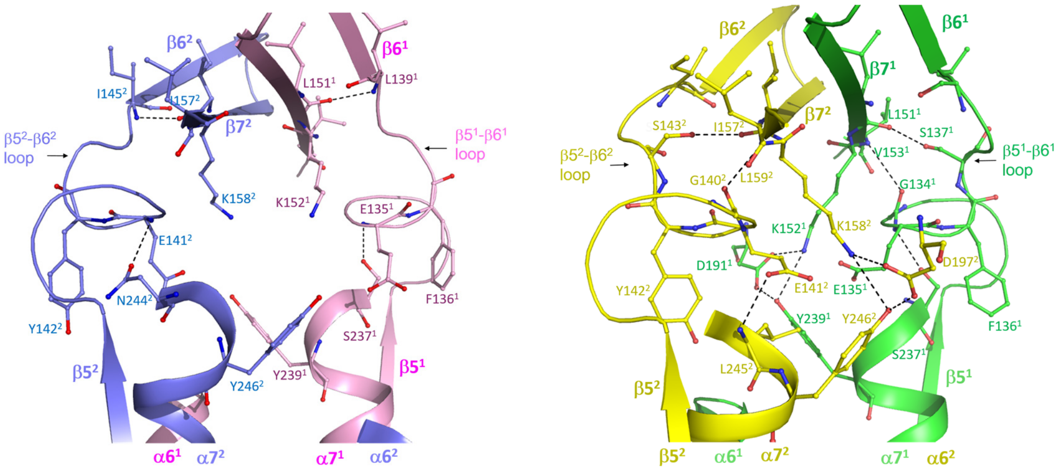

$\beta6^2$
$\beta6^1$
$I145^2$
$I157^2$
$L151^1$
$L139^1$
$\beta7^2$
$\beta5^2$-$\beta6^2$ loop
$\beta5^1$-$\beta6^1$ loop
$K158^2$
$K152^1$
$E135^1$
$E141^2$
$N244^2$
$F136^1$
$Y142^2$
$S237^1$
$\beta5^2$
$\beta5^1$
$Y246^2$
$Y239^1$
$\alpha6^1$ $\alpha7^2$ $\alpha7^1$ $\alpha6^2$
$\beta7^1$
$S143^2$
$S137^1$
$V153^1$
$G140^2$
$L159^2$
$G134^1$
$D191^1$
$D197^2$
$E141^2$ $E135^1$
$L245^2$

d

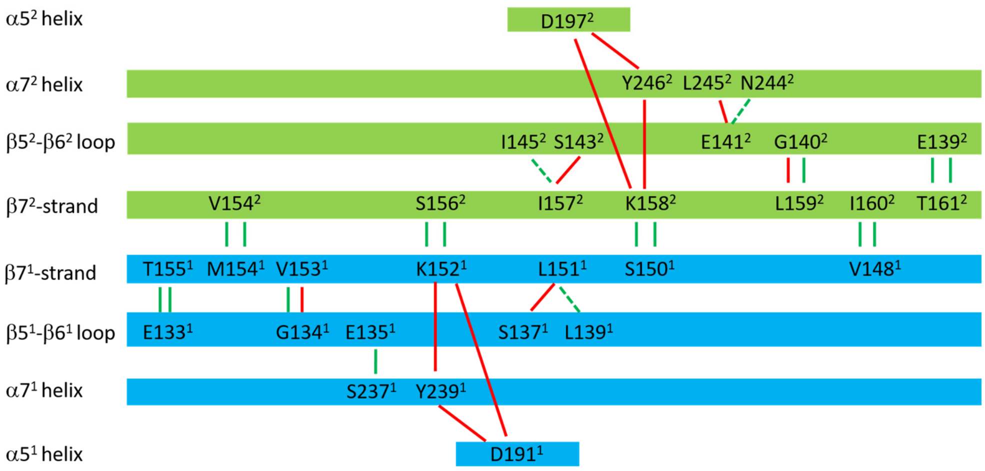

$\alpha5^2$ helix
$D197^2$
$\alpha7^2$ helix
$Y246^2$ $L245^2$ $N244^2$
$\beta5^2$-$\beta6^2$ loop
$I145^2$ $S143^2$
$E141^2$ $G140^2$
$E139^2$
$\beta7^2$-strand
$V154^2$ $S156^2$ $I157^2$ $K158^2$ $L159^2$ $I160^2$ $T161^2$
$\beta7^1$-strand
$T155^1$ $M154^1$ $V153^1$ $K152^1$ $L151^1$ $S150^1$ $V148^1$
$\beta5^1$-$\beta6^1$ loop
$E133^1$ $G134^1$ $E135^1$ $S137^1$ $L139^1$
$\alpha7^1$ helix
$S237^1$ $Y239^1$
$\alpha5^1$ helix
$D191^1$
